# CRISPR/Cas-mediated activation of genes associated with inherited retinal dystrophies in human cells for diagnostic purposes

**DOI:** 10.1172/jci.insight.189615

**Published:** 2025-09-30

**Authors:** Valentin J. Weber, Alice Reschigna, Maximilian J. Gerhardt, Thomas Heigl, Klara S. Hinrichsmeyer, Sander van den Engel, Dina Y. Otify, Zoran Gavrilov, Frank Blaser, Isabelle Meneau, Christian Betz, Hanno J. Bolz, Martin Biel, Stylianos Michalakis, Elvir Becirovic

**Affiliations:** 1 Laboratory for Retinal Gene Therapy, Department of Ophthalmology, University Hospital Zurich, University of Zurich, Zurich, Switzerland.; 2 Department of Ophthalmology, LMU University Hospital, LMU Munich, Munich, Germany.; 3 Department of Pharmacy – Center for Drug Research, LMU Munich, Munich, Germany.; 4 Department of Ophthalmology, University Hospital Zurich, Zurich, Switzerland.; 5 Bioscientia Human Genetics, Bioscientia Institute of Medical Diagnostics, Ingelheim, Germany.; 6 Institute of Human Genetics, University Hospital Cologne, Faculty of Medicine, University of Cologne, Cologne, Germany.

**Keywords:** Genetics, Ophthalmology, Diagnostics, Genetic diseases, RNA processing

## Abstract

Many patients suffering from inherited diseases do not receive a genetic diagnosis and are therefore excluded as candidates for treatments, such as gene therapies. Analyzing disease gene transcripts from patient cells holds potential for detection and interpretation of causative variants, but may be complicated by unavailability of affected tissue and/or lack of expression of the respective genes in blood or other easily accessible tissues. Using CRISPR/Cas-mediated transcriptional activation (CRISPRa), we developed a robust and efficient approach to activate genes in skin-derived fibroblasts and in freshly isolated peripheral blood mononuclear cells (PBMCs) from healthy individuals. This approach was successfully applied to blood samples from patients with inherited retinal dystrophies (IRDs). We were able to efficiently activate several IRD genes and detect their transcript isoforms using different diagnostically relevant methods such as RT-(q)PCR and long- and short-read RNA sequencing. CRISPRa-mediated transcriptional activation in PBMCs and fibroblasts will contribute to closing the critical gap in the genetic diagnosis of patients with IRD or other inherited diseases.

## Abstract 2

Gene activation of disease-related genes in acutely isolated human cells enables new diagnostic approaches for inherited (retinal) diseases.

## Graphical abstract

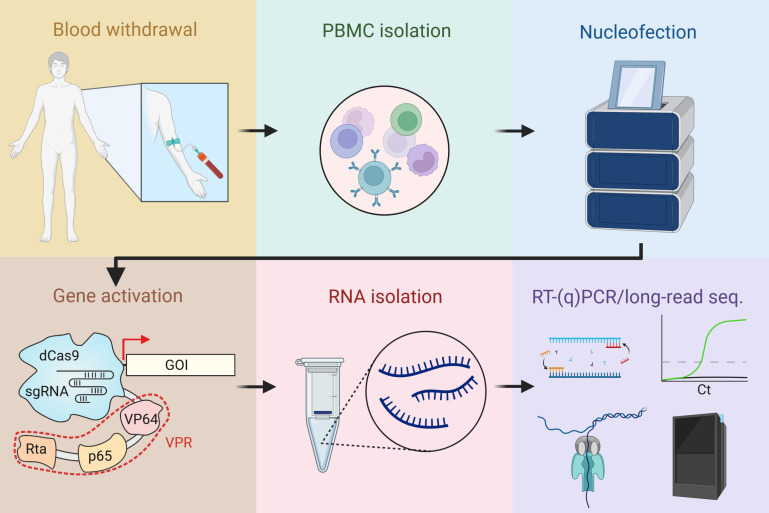

## Introduction

Even in developed countries with modern molecular diagnostic equipment, up to 50% of patients with genetic diseases receive no or insufficient genetic diagnosis (1). This excludes patients from established treatments and participation in clinical trials for novel therapies.

High-throughput next-generation sequencing (NGS) technologies, such as whole-genome sequencing (WGS) or whole-exome sequencing (WES), have facilitated the diagnostics of genetic diseases. Both WGS and WES, however, have key limitations. WES does not cover the noncoding regions (e.g., intronic sequences apart from exon-intron junctions, promoters, or other regulatory transcriptional elements), which are crucial for mRNA stability and processing. WGS is more expensive and requires extensive data storage capacities and additional bioinformatic tools for data interpretation (2, 3). Even if potentially disease-causing mutations can be identified in coding or noncoding regions of candidate genes using WGS, experimental validation of how their impact on the mRNA level is inevitable but laborious using currently available techniques, e.g., minigene assays (4) or patient-derived induced pluripotent stem cell (iPSC) models. Single-nucleotide variants can affect mRNA processing via different mechanisms. Firstly, they may alter pre-mRNA splicing (5). The potential impact of variants on splicing is usually assessed with standard splice prediction software (6, 7), but these predictions are often not reliable and may be false positive or false negative. Many false-negative splicing mutations are classified as missense variants or as silent exonic variants, especially if distant from splice junctions (8, 9). However, such point variations may affect regulatory splice elements and disrupt splicing, and should therefore be routinely tested at the transcript level, preferably in patient cells (10).

There is an unmet need for techniques enabling straightforward detection and investigation of disease-associated mutations at the transcript level. The most convenient method to analyze the transcripts of the corresponding genes is to use patients’ tissue samples. Lack of expression in tissues that can routinely be obtained from the patients (leukocytes from peripheral blood, fibroblasts from skin biopsies) represents a frequent hurdle, in particular in case of inherited retinal dystrophy (IRD) genes. The expression of some IRD genes is largely restricted to the retina, a tissue that cannot be considered for diagnostic biopsies. We therefore used CRISPR activation (CRISPRa) in acutely isolated cells (CATALYTEC) to analyze single or multiple genes associated with IRDs.

## Results

To provide proof of principle for CATALYTEC, we initially focused on the IRD genes *ABCA4*, *RPE65*, *MYO7A*, and *USH2A* for several reasons: (a) These genes are frequently mutated in the corresponding diseases: *ABCA4*, Stargardt disease (STGD1); *RPE65*, Leber congenital amaurosis (LCA) and retinitis pigmentosa (RP); *USH2A*, RP and Usher syndrome (USH). (b) Three of these genes (*USH2A*, *MYO7A*, and *ABCA4*) are very large, complicating the identification of potentially pathogenic mutations, particularly those located in noncoding regions. (c) For *RPE65*-associated IRD, an approved AAV gene supplementation therapy (voretigene neparvovec) is available. Improved genetic testing could help to assess pathogenicity of novel *RPE65* variants and thus qualify affected patients for treatment.

**Figure 1:**
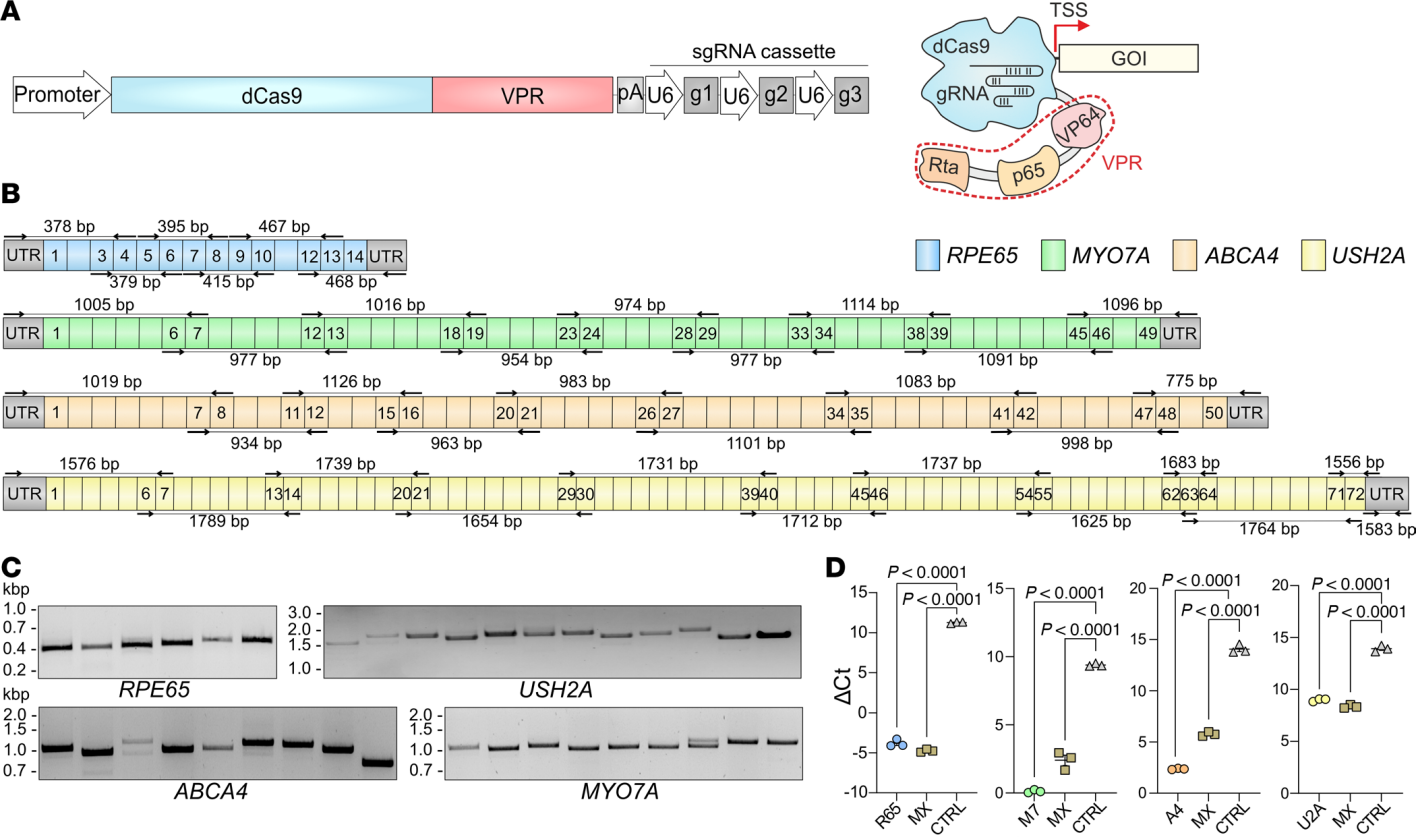
Transcriptional activation of IRD genes in transfected HEK293T. (**A**) Scheme depicting the expression plasmid and the corresponding dCas9-VPR protein bound to a gene of interest (GOI). dCas9-VPR, driven by either the CMV or SFFV promoter, was combined with a cassette expressing sgRNAs, targeting *RPE65*, *ABCA4*, *MYO7A*, or *USH2A*. For simultaneous gene activation, multiple sgRNA cassettes were combined. pA, poly(A) signal. (**B**) Primer design for RT-PCR analysis. Primer binding sites (black arrows) and PCR product lengths in base pairs (bp) are shown in the schematic representation of the transcripts. Colored boxes represent numbered exons of the respective genes. UTR, untranslated region. (**C**) RT-PCR result for each gene transcript after transcriptional activation in HEK293T cells. kbp, kilobase pairs. (**D**) RT-qPCR result for each gene activation (*n* = 3). MX, multiplexed gene transcriptional activation by combination of multiple sgRNA cassettes. R65, *RPE65*; M7, *MYO7A*; A4, *ABCA4*; U2A, *USH2A*. Values are shown as mean ± SEM. Statistics/multiple comparisons were calculated with 1-way ANOVA and Dunnett’s test.

Our initial gene activation experiments were performed and optimized in HEK293T cells transfected with cassettes expressing nuclease-deficient dCas9-VPR, one of the most effective CRISPRa systems (11, 12), in combination with single guide (sg) RNAs targeting the transcriptional start site (TSS) region of the respective genes (Figure 1A). We were able to identify sgRNAs for efficient activation of each of these genes. Multiplexing experiments demonstrated that the combination of multiple sgRNA cassettes resulted in simultaneous activation of all genes without any substantial loss of activation efficiency (Figure 1D). Using RT-PCR, we could amplify all fragments covering the entire coding region and parts of the untranslated regions (Figure 1, B and C, and Supplemental Figure 1; supplemental material available online with this article; https://doi.org/10.1172/jci.insight.189615DS1). The identity of all bands was confirmed by Sanger sequencing.

In the first attempts to establish a robust and simple protocol for the transfection of PBMCs and skin fibroblasts from healthy individuals, we tested various commonly used transfection techniques (e.g., calcium phosphate, lipofection, and electroporation). For most of these approaches, however, we observed no or weak expression of the transfected GFP reporter gene.

Since lentiviral vectors (LVs) have been shown to efficiently transduce nondividing cells (13), we investigated the efficacy of gene activation upon transduction with LVs. In our experiments, using dual LVs, each expressing GFP or dsRed, we obtained high cotransduction efficiencies for human skin fibroblasts and PBMCs (Supplemental Figure 2, A and B). Surprisingly, the dual LV approach for gene transcriptional activation in transduced HEK293T, fibroblasts, and PBMCs led to a relatively weak transcriptional activation of *ABCA4*, despite numerous optimization steps (e.g., use of high multiplicity of infections [MOIs], different promoters etc., Supplemental Figure 2, C–F). These results suggest that dCas9-VPR delivered via LVs is substantially less efficient in activating target genes compared with delivery by plasmid transfection. To elucidate the causes for the low activity of dCas9-VPR in cells transduced with the corresponding LVs, we generated a HEK293T cell line stably expressing dCas9-VPR through genomic integration of the corresponding cassette (Supplemental Figure 3A). After transduction of these cells with LVs expressing only the sgRNAs targeting the individual genes, we detected a robust transcriptional activation of *ABCA4*, *MYO7A*, and *USH2A*. A similarly high activation was also achieved in a multiplexing approach, in which all 3 genes were targeted simultaneously (Supplemental Figure 3B). Together with the results shown in Supplemental Figure 2, this indicates that the efficiency of transcriptional activation is not compromised by LV-mediated delivery of sgRNAs, excluding a general impact of LVs on gene activation. A possible explanation for our observation of low-level gene activation in all cell types upon LV-based administration of dCas9-VPR is that dCas9-VPR is not well tolerated by LV vectors due to its size (5.8 kb). To test this hypothesis, we used a shorter variant of this CRISPRa system, dCas9-VPRmini (Supplemental Figure 3C) (14). However, this approach also did not lead to increased activation efficiency compared with full-length dCas9-VPR (Supplemental Figure 3D).

From this set of unfavorable results, we concluded that the LV-based approach in combination with dCas9-VPR–mediated transcriptional activation of genes is not suitable for diagnostic purposes.

**Figure 2:**
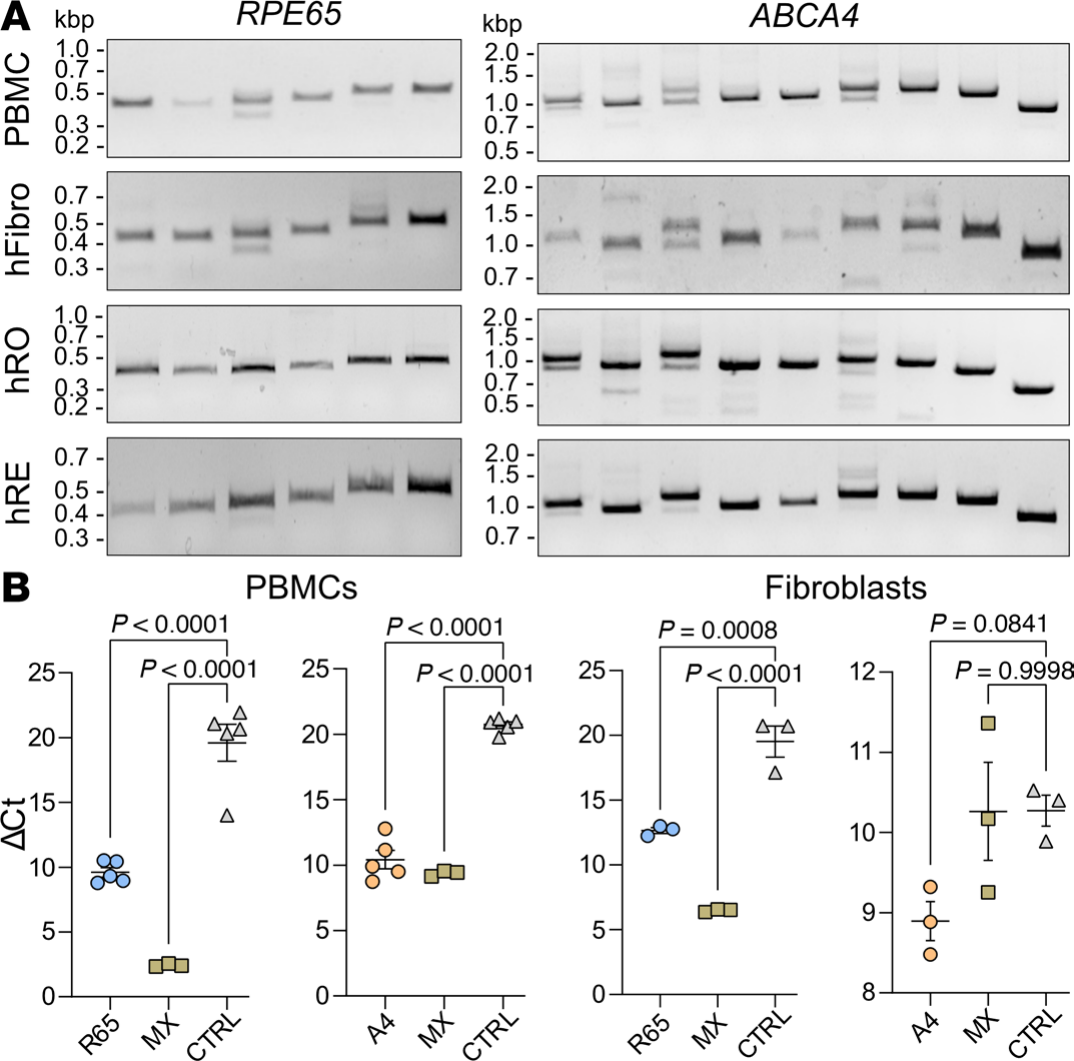
RT-(q)PCR analyses of transcriptionally activated genes in human PBMCs and fibroblasts. (**A**) RT-PCR results for *RPE65* (left) and *ABCA4* (right). Transcriptionally activated PBMCs and fibroblasts are compared to human retinal organoids (hROs) and human retina (hRE) with endogenously expressed *RPE65* and *ABCA4*. (**B**) RT-qPCR results for transcriptionally activated *RPE65* (R65) and *ABCA4* (A4) in PBMCs (left; *n* = 5) and fibroblasts (right; *n* = 5). MX, multiplexed transcriptional activation of both genes (*n* = 3). Values are given as mean ± SEM. Statistics/multiple comparisons were calculated using 1-way ANOVA and Dunnett’s test.

Achieving reasonable levels of transcriptional activation for genes of interest in PBMCs or skin fibroblasts from healthy individuals is a prerequisite for establishing a robust protocol for diagnostic purposes in patient cells. So far, although we initially obtained high activation efficiencies for the individual IRD genes in transfected HEK293T cells, none of the previously described approaches has reached this goal. In another attempt, we used nucleofection for transferring sgRNA and transgene plasmids into our primary target cells. Nucleofection of a GFP-encoding cassette resulted in robust expression of this reporter in both cell types (Supplemental Figure 4A). Next, we nucleofected the dCas9-VPR expression cassette together with sgRNAs targeting *MYO7A*, *USH2A*, *ABCA4*, or *RPE65*. Using RT-qPCR, we observed high activation efficiencies for *ABCA4* and *RPE65*. Additionally, we were able to detect the entire coding and untranslated regions using RT-PCR (Figure 2, A and B, and Supplemental Figure 5). By comparison, we found that *MYO7A* is already endogenously expressed in human skin fibroblasts and in PBMCs, respectively, in sufficient amounts to be detected by RT-PCR. Nevertheless, after transcriptional activation a moderate increase in *MYO7A* expression was detectable via RT-qPCR (Supplemental Figure 4, B and C). For *USH2A*, we could only detect RNA corresponding to exons 1–7, 45–55, and the 3′-UTR (Supplemental Figure 4, D and E). To optimize transcriptional activation of *USH2A* in PBMCs, we replaced dCas9-VPR with p65-HSF1-Suntag, another effective CRISPRa module (12). However, similar to dCas9-VPR, p65-HSF1-Suntag led to an efficient *USH2A* activation in HEK293T cells, but no expression of relevant transcripts was detectable in PBMCs (Supplemental Figure 6C).

As shown previously, inhibition of nonsense-mediated mRNA decay (NMD) could increase the expression of activated transcripts in patient-derived cells, such as skin fibroblasts (15, 16). Indeed, using cycloheximide to inhibit NMD in PBMCs, we were able to detect larger parts of the *USH2A* transcript covering exons 1–46 and the 3′-UTR (Supplemental Figure 6, A and B).

One possible explanation for the discrepancy in efficiency of *USH2A* activation between HEK293T cells and PBMCs would be that sgRNA target sites contain some nonpathogenic variants, such as single-nucleotide polymorphisms (SNPs), or small insertion and deletion (INDEL) sites. However, we found no INDELs or SNPs that overlapped with the sgRNA binding sites of the *USH2A* and *RPE65* genes. Nevertheless, multiple SNPs were identified in binding regions of sgRNAs targeting *ABCA4* and *MYO7A* (Supplemental Table 6).

So far, we have achieved efficient activation in PBMCs for 3 of the 4 genes examined, enabling detection of the entire transcript. The fact that *USH2A* could not be efficiently activated may be due to the sheer size of the gene/transcript (>800 kb/>18.9 kb). Another explanation could be that our CATALYTEC approach is less efficient in PBMCs than in HEK293T cells. To address this, we activated 2 additional genes associated with the Usher 2 subtype: *PDZD7* and *ADGRV1*, with the latter gene being comparable to *USH2A* with respect to its gene/transcript size (>600 kb/>19.5 kb). The corresponding RT-qPCR data from PBMCs show a substantial increase in gene expression for both genes. Moreover, the activation efficiency was stronger in PBMCs compared with HEK293T cells (Supplemental Figure 7), suggesting that neither the gene’s size nor the inherent inefficiency of CATALYTEC in PBMCs was the main cause of the low and incomplete activation of *USH2A* in these cells.

For *RPE65* and *ABCA4*, we detected several splice variants besides the expected RT-PCR bands encoding the full-length proteins, most of which resulted in frameshifts and premature termination codons (PTCs) (Supplemental Tables 1 and 2). The corresponding bands could represent alternative splicing that might differ in PBMCs and in the retina.

To test how the splice pattern observed in PBMCs compared to that of human retinal cells, we performed RT-PCR of RNA samples isolated from human donor retinas and human retinal organoids using the same primer pairs. We observed a very similar band pattern compared to the RT-PCR results from skin fibroblasts and PBMCs, suggesting no major differences in mRNA splicing of *ABCA4* and *RPE65* between the different cell and tissue types (Figure 2A).

**Figure 3:**
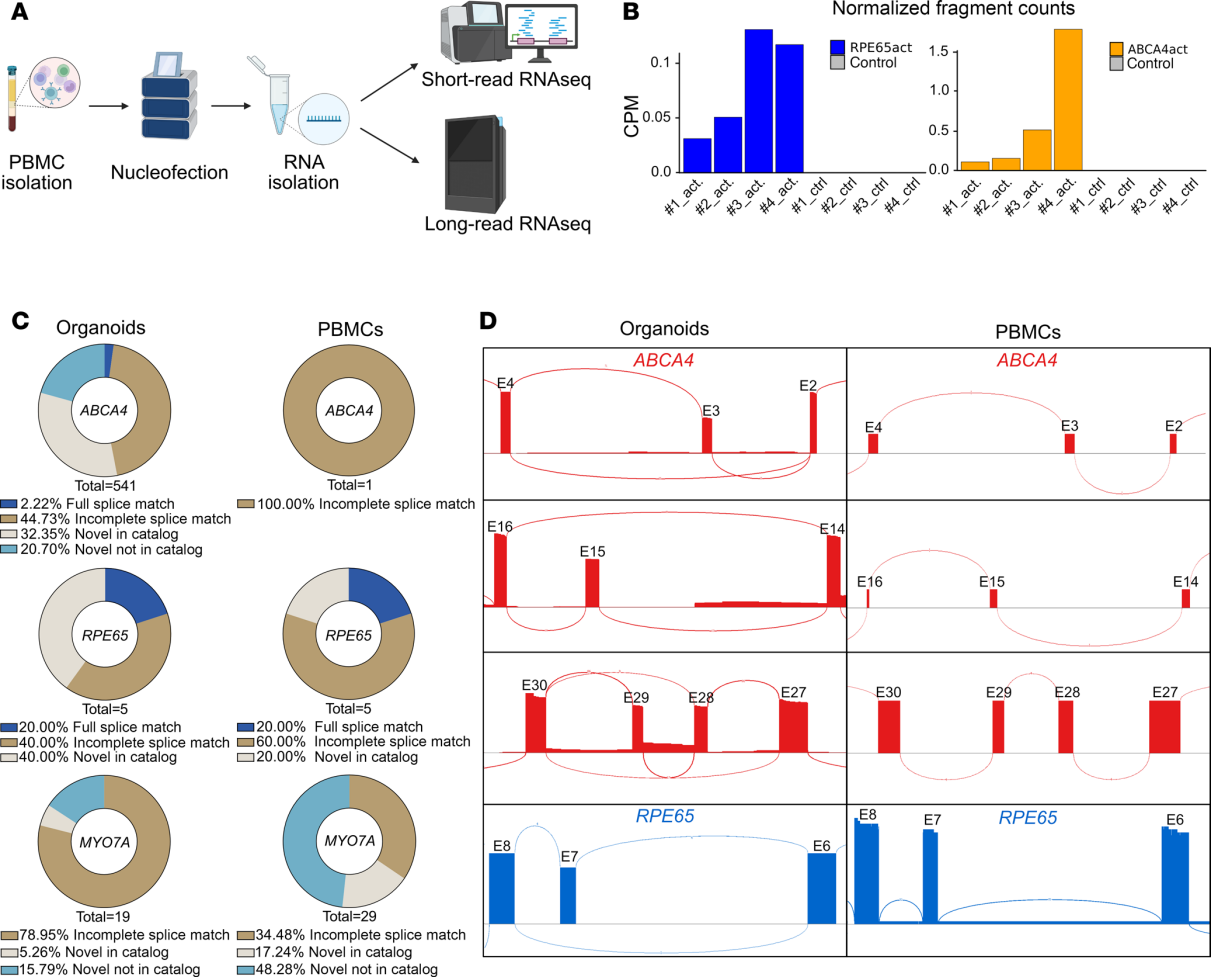
Short- and long-read RNA-Seq of transcriptionally activated PBMCs. (**A**) Workflow diagram from PBMC isolation to short- and long-read RNA-Seq. (**B**) Normalized fragment counts for *RPE65* (left, blue) and *ABCA4* (right, orange) of 4 analyzed samples (nos. 1–4) compared to untreated control samples. CPM, counts per million. (**C**) Proportion of different read categories for each *ABCA4*, *RPE65*, and *MYO7A* after long-read RNA-Seq of human retinal organoids (left) and activated PBMCs (right). (**D**) Sashimi plot for the evaluation of alternative splicing of *ABCA4* (red) and *RPE65* (blue) at respective locations found after RT-PCR and Sanger sequencing (Supplemental Tables 1 and 2). Red and blue bars represent reads covering the respective exons. Red and blue lines connecting these bars represent reads spanning the respective exon-exon junction.

Although RT-PCR is a robust method for the qualitative detection of mRNA splicing defects, it is less suitable for the reliable quantification of the results. In addition, particularly for low-abundance transcripts, it is susceptible to artificial variations, manifesting during cDNA synthesis and subsequent PCR amplification. To overcome the drawbacks of RT-PCR and to validate our results with additional methods, we analyzed RNA from PBMCs of several healthy individuals using 2 next-generation RNA sequencing (RNA-Seq) techniques: short-read RNA-Seq (Illumina) and long-read RNA-Seq (PacBio Revio) (Figure 3A). Additionally, to compare the transcript pattern of activated genes in PBMCs to their counterparts from a more native environment, we performed long-read sequencing of RNA isolated from human retinal organoids.

Short-read RNA-Seq of samples with transcriptionally activated *ABCA4* and *RPE65* showed a robust increase in gene expression for both genes in comparison to nontreated control samples (Figure 3B). Splice junction analysis of *ABCA4* demonstrated that the level of transcriptional activation achieved with CATALYTEC is in principle sufficient to analyze the different transcript isoforms (Supplemental Figure 4F). Simultaneous transactivation of *ABCA4* and *RPE65* and single transactivation of *MYO7A* led to full transcript coverage by long-read RNA-Seq, corroborating the increase in gene expression as previously determined via RT-qPCR and short-read RNA-Seq (Supplemental Figure 8A). The respective consensus sequences, constructed by clustering similar reads and eliminating potential artifacts, were used to identify isoforms of biological relevance. For *RPE65* and *MYO7A*, several full-length isoforms were identified as consensus sequences (Supplemental Figure 8B). In contrast, only 1 consensus sequence was obtained for *ABCA4*, as multiple transcript reads were excluded in the filtering process.

Long-read RNA-Seq of human retinal organoids resulted in the identification of more than 500 sequence reads within the *ABCA4* gene, of which more than 95% were classified as incomplete splice matches or novel in catalog. Only a few reads were sequenced for *RPE65* (*n* = 5) and *MYO7A* (*n* = 19; Figure 3C). Sequencing of transcriptionally activated PBMCs resulted in only 1 read within the *ABCA4* gene. For *MYO7A* and *RPE65*, long-read sequencing showed similar or higher transcript abundance in PBMCs compared with human retinal organoids (Figure 3C).

Following long-read RNA-Seq of human retinal organoids, we were able to confirm alternative splicing of *ABCA4* and *RPE65* in PBMCs, previously identified after Sanger sequencing of RT-PCR amplicons (Supplemental Tables 1 and 2, and Figure 3D).

**Figure 4:**
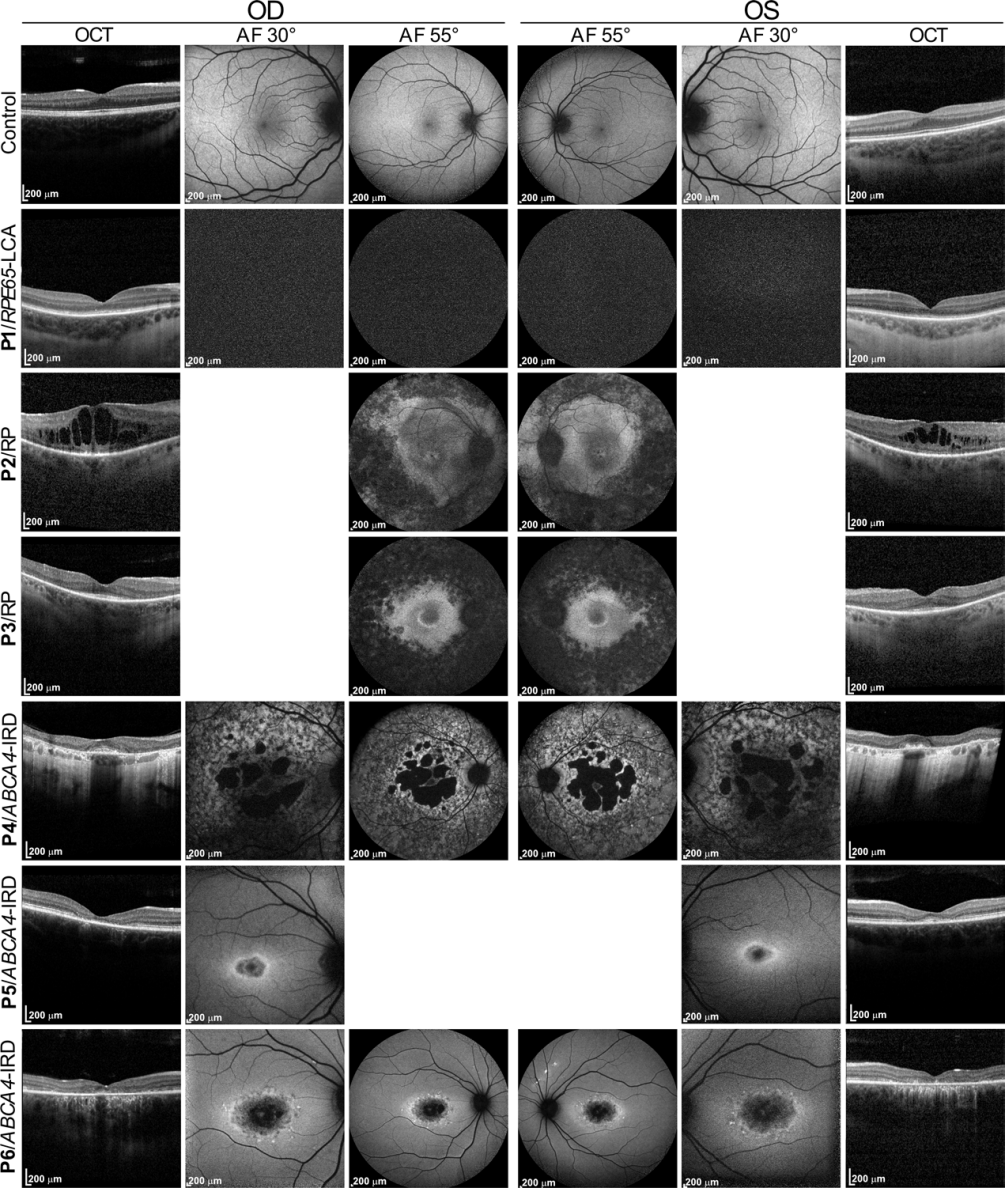
Clinical phenotype of patients. The top panel shows optical coherence tomography (OCT), 30° and 55° fundus autofluorescence (AF) images of a healthy subject (control). The next 3 lower panels show the corresponding images of 3 individuals (P1–P3) with confirmed *RPE65*-associated retinal disease (*RPE65*-LCA and RP). P1 shows no AF due to severe *RPE65* deficiency. The last 3 panels show the corresponding images of 3 individuals (P4–P6) with confirmed *ABCA4-*associated retinal disease (STGD1). OD, oculus dexter. OS, oculus sinister.

Finally, we applied the described CATALYTEC protocol to 1 patient with confirmed biallelic *RPE65*-associated LCA (P1), 2 patients with the clinical phenotype of rod-cone dystrophy (RP, P2 and P3) but unclear molecular genetic diagnosis, and 3 patients with the clinical diagnosis of *ABCA4*-associated STGD (P4–P6) (Table 1 and Figure 4).

All patients, except for P3, had previously undergone molecular genetic testing, of whom only P1 and P4 had a definite molecular genetic diagnosis (compound heterozygosity), which was confirmed by segregation analysis.

P1 is a 6-year-old male who has been severely visually impaired from birth and received a genetic diagnosis of *RPE65*-associated LCA at the age of 5 years. He was treated with voretigene neparvovec at the age of 6 years (17). P2 and P3 are 2 female siblings who first experienced impaired vision (night blindness and, in the further course, visual field restriction) in their early 20s with the clinical diagnosis of rod-cone dystrophy (RP) lacking a definite genetic diagnosis. In 2013, panel sequencing of 42 IRD genes in P2 revealed 3 heterozygous variants of unknown significance in *RPE65*, *CRB1*, and *RP1L1*, respectively (Table 1). WES performed in 2021 did not identify any additional candidate variants. P4 is a 49-year-old female who first experienced visual deterioration at the age of 33 years representing late-onset STGD with pronounced macular atrophy and foveal sparing (Figure 4). P5 is a 28-year-old female and P6 is her 31-year-old brother, both diagnosed with macular dystrophy at the ages of 19 and 21, respectively.

Potentially pathogenic compound heterozygous mutations in *ABCA4* and *RPE65* were found in all STGD1 patients (P4–P6) and in P1, respectively (Table 1).

**Figure 5:**
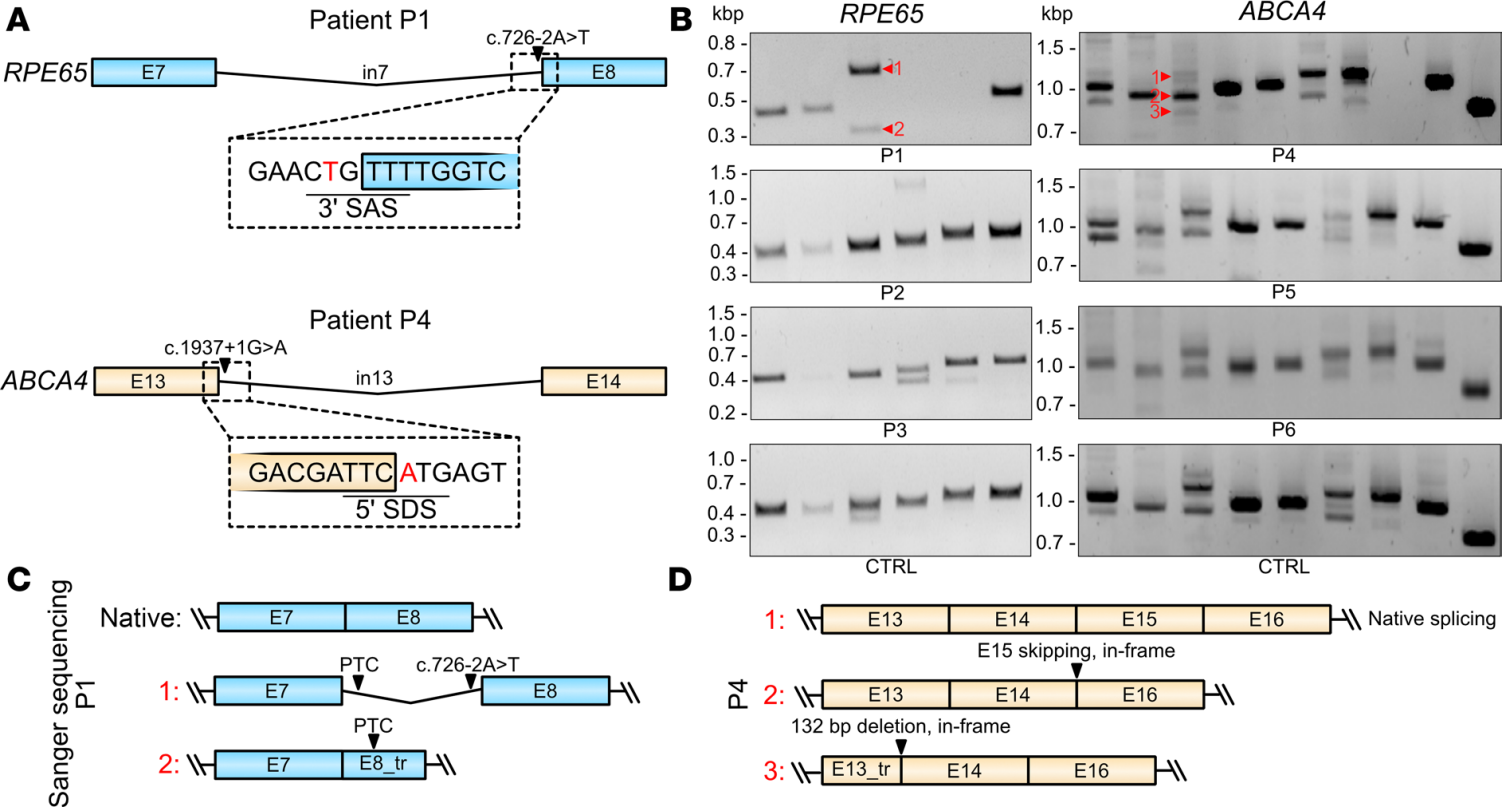
*RPE65* and *ABCA4* transcript analysis in patient samples. (**A**) Scheme highlighting the position of the c.726-2A>T mutation in *RPE65* (patient P1; blue) and c.1937+1G>A mutation in *ABCA4* (patient P4; orange). Colored boxes represent exons, the line in between represents the intron. (**B**) RT-PCR results for patients P1–P6 in comparison to a healthy human control sample (CTRL). (**C**) Scheme summarizing Sanger sequencing result of the numbered bands 1 and 2 of sample P1 shown in **B**. PTC, premature termination codon. (**D**) Scheme summarizing the results of RT-PCR analysis of the numbered bands 1–3 from sample P4, as indicated in **B**.

We performed the CATALYTEC on PBMCs of these patients and analyzed the RNA by RT-PCR. P1 carries c.11+5G>A within the extended splice donor consensus sequence of exon 1 in the *RPE65* gene. According to RT-PCR analysis, this mutation does not severely affect mRNA splicing at that position (Figure 5B and Supplemental Figure 9). For the second mutation affecting the canonical consensus sequence of the splice acceptor site in intron 7 (c.726-2A>T, Figure 5A), RT-PCR analysis revealed a clear difference in the band pattern compared with the unaffected control sample, experimentally confirming the splicing divergence. Sanger sequencing of these bands revealed partial skipping of exon 8 and complete inclusion of intron 7 (Figure 5, B and C).

The c.1937+1G>A mutation of P4 disrupts the splice donor site of exon 13 in *ABCA4* (Figure 5A). A previous case study has associated this mutation with severe phenotypes, including STGD and macular degeneration (18). A recent publication reported that it causes aberrant splicing by activating cryptic splice donor sites within exon 13 and intron 13, ultimately resulting in a 132 bp in-frame deletion (19). This conclusion was based on data obtained with a minigene assay. Potential consequences at the protein level, such as p.[(Y.603_S646del, F647*)], have not yet been experimentally validated. For this mutation, we visually detected an increased ratio of alternative exon 15 splicing in comparison with healthy control samples in RT-PCR (Figure 5, B and D, and Supplemental Figure 9). As described in Table 1, this alternatively spliced *ABCA4* variant leads to an in-frame skipping of exon 15. However, the significance of this deletion for ABCA4 protein expression, stability, and function remains unclear and is outside the focus of this study. For all other patients carrying potentially pathogenic mutations in IRD genes analyzed herein, no obvious effects on splicing were detected (Figure 5B).

Taken together, these results suggest that our CATALYTEC is in principle suitable for the detection and quantification of splicing mutations in patients’ PMBCs.

## Discussion

Here, we established CATALYTEC, a robust and simple approach for CRISPRa-based activation of genes in human PBMCs and skin fibroblasts for routine diagnostic purposes.

CATALYTEC fulfills various criteria for use in diagnostics: (a) It is simple and easy to implement. (b) It may indicate mutations that have either escaped detection due to technical limitations. (c) It can be used to validate the proposed pathogenicity of detected mutations or adjust the categorization seemingly neutral changes (e.g., silent exonic variants). (d) It is without further modifications transferable to other genetic disorders where biopsy of affected tissues is not possible.

In recent studies, similar approaches were developed to activate the genes associated with various inherited diseases, including *CRB1* (RP) and *USH2A*, in human skin fibroblasts (15, 16, 20). Compared with these studies, our method offers additional key advancements. We demonstrate that dCas9-VPR can effectively activate disease-associated genes in PBMCs readily isolated from patient blood samples. Skin fibroblasts have the disadvantage that their isolation requires skin punching, which is more elaborate and associated with reduced patient compliance. Therefore, genetic testing of human skin fibroblasts is less suitable for broad routine diagnostics compared to PBMCs. Additionally, the isolation and cultivation of skin fibroblasts is more time consuming. With our CATALYTEC approach, we achieved a turnaround time from blood collection to first RT-PCR results of 48–72 hours, ultimately increasing the convenience for patients. Under the conditions used herein, we could not achieve sufficient activation of *USH2A* in PBMCs to cover all coding regions of the corresponding transcript. Given that this gene could be sufficiently activated in HEK293T cells in this study and recently also in human skin fibroblasts, we assume that the chromatin accessibility of *USH2A* is cell type dependent.

We have applied CATALYTEC to several common genes associated with IRDs, a heterogeneous group of genetic diseases affecting the retina. In particular, we have proven the ability of our approach to activate large genes like *ABCA4* and *MYO7A*. This is of great importance, as deep-intronic mutations in large genes are not covered by WES or, when identified by WGS, cannot be correctly interpreted in terms of their potential impact on mRNA splicing.

Our data show that the level of transcriptional activation obtained with CATALYTEC is high enough to be combined with the most commonly used readout methods (RT-PCR, RT-qPCR, short- and long-read RNA-Seq). RT-PCR analysis provided the first evidence that CATALYTEC can be used to detect pathogenic splice mutations in PBMCs of IRD patients. Additionally, we show that short- and long-read RNA-Seq can be used to analyze the expression and alternative splicing of transcriptionally activated genes in PBMCs. However, a meaningful and substantial result for diagnostic purposes necessitates a more detailed analysis of isoforms, which could be achieved through higher coverage of the respective target genes.

We demonstrate that the splicing patterns of activated IRD genes in human PBMCs and fibroblasts are very similar to those observed in human retinas and retinal organoids. Yet, we cannot exclude the possibility that other activated genes in PBMCs might have different splicing patterns than in the cells in which these genes are naturally expressed. In addition, rare tissue-specific splicing events may have been missed by RT-PCR. More detailed investigations using short- and long-read sequencing of the human retina and organoid transcriptomes could provide valuable insights in this regard.

An attractive alternative to PBMCs would be an application of the CATALYTEC protocol in buccal epithelial cells, as their collection is technically even simpler and less invasive than blood sampling. It remains to be seen whether this goal can be achieved using CRISPRa or other methods for introducing DNA or RNA into these cells.

In summary, we provide a proof of concept for a CRISPRa-based approach to activate IRD genes in PBMCs in sufficient quantities. The resulting transcripts can be detected and quantified using standard methods and analyzed for structural variants and splicing defects. The CATALYTEC method is universally applicable, can be easily adapted to other target genes, and could help closing important gaps in the diagnosis of inherited (retinal) diseases.

## Methods

### Sex as a biological variable.

Our study examined men and women, as sex should not be an exclusion criterion due to the diagnostic background of the study. Sex was not considered as a biological variable.

### Optical coherence tomography and autofluorescence imaging.

Retinal cross sections were obtained with spectral domain optical coherence tomography (SD-OCT) using the Heidelberg Spectralis OCT (Heidelberg Engineering). Autofluorescence (488 nm) was obtained using the same device.

### Plasmids.

The pcDNA3.1-CMV-dCas9-VPR and plasmids for LV production (pMDL, pRSVRev, and pMD2.G) were obtained from Addgene (catalog 63798, 12251, 12253, and 12259). Expression of dCas9-VPR in pcDNA3.1 vectors was driven by the cytomegalovirus (CMV) promoter. In lentiviral CRISPRa systems, the dCas9-VPR expression was induced by the CMV, spleen focus-forming virus (SFFV), or elongation factor 1α (EF1α) promoter.

sgRNA cassettes were synthesized (Azenta, IDT) and inserted using standard cloning techniques. Sequences are shown in Supplemental Table 3. All plasmids were sequenced before use (Eurofins Genomics/Microsynth).

### Cell culture.

HEK293T cells (DMSZ, ACC635) were maintained in high-glucose DMEM (Thermo Fisher Scientific) supplemented with 10% FBS (Superior, Sigma-Aldrich) and 1% penicillin/streptomycin (P/S, Thermo Fisher Scientific) at 37°C, 10% CO_2_.

Human skin fibroblasts (adult, Sigma-Aldrich, 106-05A) were cultured in DMEM (low glucose) supplemented with 10% FBS and 1% P/S at 37°C, 5% CO_2_.

PBMCs, freshly isolated from human whole blood, were cultured in RPMI-1640 (Thermo Fisher Scientific) supplemented with 10% FBS at 37°C, 5% CO_2_.

### Collection and stimulation of PBMCs.

PBMCs were isolated through density centrifugation of the collected whole blood. The isolated buffy coat was washed with PBS and resuspended in RPMI-1640, supplemented with 10% FBS and 1× phytohemagluttinin-L (PHA-L; Thermo Fisher Scientific). The cells were incubated at 37°C and 5% CO_2_ for 20 hours before being used for nucleofection.

### Inhibition of NMD.

Inhibition of NMD was initiated 24 hours after nucleofection by adding 200 mg/mL (final concentration) of cycloheximide (Sigma-Aldrich) to the culture medium. After 24 hours, the cells were harvested and used for RNA isolation.

### Isolation of human retinas.

Human donor eyes without cornea and lens were received from the eye bank of the University Hospital Zurich in ice-cold PBS. Retina and eyecups were separately flat mounted after making 4 incisions. Retina samples were collected from the nasal periphery and the macular region. The isolated retina was snap-frozen and stored at –80°C until further processing.

### Transfection of cell lines and primary cells.

HEK293T were transfected with either Xfect (Takara Bio) or Lipofectamine 3000 (Invitrogen) according to the manufacturers’ instructions and harvested 48 hours after transfection.

PHA-L–stimulated PBMCs (2.0–2.5 × 10^7^ cells per reaction) were nucleofected with the P3 Primary Cell 4D-Nucleofector Kit (Lonza). After application of program EO-115, 500 μL of pre-equilibrated culture medium was added to the cuvette and the suspension was immediately transferred to a pre-equilibrated 12-well plate. Nucleofected PBMCs were incubated for 24 hours at 37°C, 5% CO_2_.

For nucleofection of fibroblasts (2 × 10^6^ cells per reaction) the P2 Primary Cell 4D-Nucleofector Kit (Lonza) was used. After application of program CZ-167, 500 μL of pre-equilibrated culture medium was added to the cuvette and the suspension was immediately transferred to a pre-equilibrated 6-well plate. Nucleofected fibroblasts were incubated for 24 hours at 37°C, 5% CO_2_.

### RNA isolation.

HEK293T and fibroblasts were washed with PBS and lysed with RLT Plus buffer (Qiagen) supplemented with 10 μL/mL β-mercaptoethanol (Carl Roth). The cell lysate was transferred into safe-lock tubes (Eppendorf) and homogenized with a mixer mill (Retsch) at 30 Hz for 1 minute. After centrifugation, RNA was isolated according to the manufacturer’s protocol of the RNeasy Plus Mini Kit (Qiagen).

For PBMCs, the cell were pelleted and lysed with RLT plus buffer supplemented with 10 μL/mL β-mercaptoethanol. Subsequently, the lysate was added to QIAshredder homogenization columns (Qiagen) and centrifuged according to the manufacturers’ protocol. After homogenization, the RNeasy Plus Mini Kit was used to isolate the RNA. Snap-frozen retinas were lysed in buffer RLT supplemented with 10 μL/mL β-mercaptoethanol. The retinas were homogenized through a 21-gauge needle. Afterwards, the RNA was isolated with the RNeasy Plus Mini Kit.

RNA isolation from human retinal organoids was done with the Direct-zol DNA/RNA miniprep kit (Zymo) according to the manufacturer’s instructions. Two human retinal organoids at the same age were pooled to obtain enough RNA for subsequent experiments.

To avoid gDNA contamination, an on-column DNase I digestion (Qiagen) was performed for every RNA isolation.

### Two-step RT-PCR.

First-strand cDNA for 2-step RT-PCR analysis of HEK293T, PBMCs, and fibroblasts was produced with Maxima H Minus Reverse Transcriptase (Thermo Fisher Scientific) according to the manufacturer’s protocol. First-strand cDNA from retinal RNA was synthesized with the M-MLV reverse transcriptase (Promega).

Subsequent second-strand synthesis and PCR amplification was performed with the Q5 Hot Start High Fidelity Polymerase (New England Biolabs). Primers used for amplification are listed in Supplemental Table 4. Results were visually analyzed through agarose gel electrophoresis (1% w/v). Amplified bands were isolated with the QIAquick Gel Extraction Kit (Qiagen) and sent for Sanger sequencing (Microsynth AG, Eurofins).

### RT-qPCR.

For quantitative real-timer PCR, RNA was reverse transcribed into cDNA with the RevertAid First Strand cDNA synthesis Kit (Thermo Fisher Scientific). The SYBR Green PCR Master Mix (Thermo Fisher Scientific) was used to prepare the samples according to the manufacturer’s instructions. For amplification and analysis, the MicroAmp Fast Optical 96-Well Reaction Plate and QuantStudio 3 RT-PCR system and software (Thermo Fisher Scientific) were used. Expression levels were normalized to *ALAS1*. Primers used for RT-qPCR analysis are listed in Supplemental Table 5.

### Lentivirus production.

For production of LVs, HEK293T were transfected with pMDL, pRSVRev, and pMD2.G as well as the transgene plasmid plasmids via calcium phosphate method. The transfected cells were incubated for 48 hours at 37°C, 10% CO_2_. Subsequently, the cell culture medium was collected and filtered with a 0.45 μm filter unit (VWR). The cells were supplied with fresh culture medium and incubated for additional 24 hours. The filtered medium was centrifuged at 50,000*g* and 17°C for 2 hours (Beckman Coulter). The pellet was suspended in 250 μL HBSS (Thermo Fisher Scientific) and stored at 4°C overnight. The described procedure was repeated with the cell culture media, added the day before. The first and second harvest were combined and concentrated with a sucrose cushion centrifugation at 45,000*g*, 17°C for 2 hours. The resulting pellet was suspended in 70 μL HBSS and mixed for 45 minutes at 1400 rpm. Final aliquots of 5 μL were stored at –80°C.

Titer determination was performed with a RT-qPCR Lentivirus Titer Kit (Applied Biological Materials Inc.) according to the manufacturer’s instructions.

### Generation of human retinal organoids.

Human retinal organoids were differentiated from the human-derived iPSCs (F49B7, provided by Botond Roska, Institute for Molecular and Clinical Ophthalmology Basel [IOB], Basel, Switzerland). Pluripotency markers and germ layer differentiation potential were determined as previously described (21). iPSCs were seeded on Matrigel-coated 6-well plates (Corning) and cultured in mTeSR plus medium (STEMCELL Technologies) at 37°C, 5% CO_2_. The iPSCs were passaged using 0.5 mM EDTA (pH 8.0, Thermo Fisher Scientific).

Differentiation of iPSCs into human retinal organoids was performed according to the protocol developed by Kim et al. (22), with some modifications. All organoids at the different maturation stages were cultured in a humidified incubator at 37°C, 5% CO_2_.

On day 0, the iPSCs were dissociated using 0.5 mM EDTA. The aggregates were suspended in cold Matrigel (GFR, Corning) and incubated at 37°C for 20 minutes. Afterwards, the iPSC/Matrigel aggregates were dispersed in neural induction medium (DMEM/F12 with neurobasal medium [1:1] supplemented with 1% B27 [with vitamin A supplement], 0.5% N-2 supplement, 0.1 mM β-mercaptoethanol, 2 mM GlutaMax, and 1% P/S [all Thermo Fisher Scientific]) and cultivated in ultra-low-adherence 6-well plates (Costar, Corning). On day 5, floating cysts were seeded on Matrigel-coated 6-well plates. On day 15, cysts were detached by adding Dispase (0.5 mg/mL in DMEM/F12; STEMCELL Technologies), washed with DMEM/F12, and further cultured in retinal differentiation medium (DMEM/GlutaMax supplemented with F12 nutrition mix [3:1], 2% B27 [without vitamin A), 1% nonessential amino acids [NEAAs], and 1% P/S). On day 25, the immature retinal organoids were transferred to retinal maturation medium (DMEM/GlutaMax supplemented with F12 nutrition mix [3:1], 8% FBS, 2% B27 [without vitamin A], 1% NEAAs, 1% antibiotic/antimycotic, and 1% 100 mM taurine [Sigma-Aldrich]).

On day 230, mature organoids were used for RNA isolation and subsequent experiments.

### Direct sequencing of patient-derived blood samples.

For the *ABCA4* mutation analysis of patient P4, exons 4, 13, and 40 were PCR amplified and directly sequenced. The obtained sequences were compared with the reference sequence NM_000350.2.

### WES of patient-derived blood samples.

Genomic DNA, isolated from collected blood samples from patients P2, P5, and P6, was fragmented, and the coding exons of the analyzed genes as well as the corresponding exon-intron boundaries were enriched using Roche/KAPA sequence capture technology (KAPA HyperExome Library) and sequenced using an Illumina NovaSeq 6000 system. The requested gene panel was extracted from the WES data. The target regions were sequenced with an average coverage of 337×. For more than 99% of the target regions, a 15-fold coverage was obtained. Putatively pathogenic differences between the wild-type sequence (human reference genome according to UCSC Genome Browser: hg19, GRCh37) and the interpreted patient’s sequence were assessed using an internally established quality system. Variants that did not pass the quality threshold were verified using conventional Sanger sequencing. Variants listed as additional, putatively relevant variants were not routinely validated. Identified variants were compared to literature and external as well as internal allele frequency databases (e.g., gnomAD). In addition, in silico analysis of the identified variants with regard to functional relevance, conservation, and splice effects was performed using bioinformatic prediction programs (e.g., SpliceAI, MaxEntScan). The variants were classified using the current ACMG guidelines (23).

WES for patient P1 was performed with the Twist Human Core Exomes combined with Twist Human RefSeq Panel (Twist Biosciences).

### Long-read WGS of patient-derived blood samples.

Genomic DNA, isolated from collected blood samples, was fragmented, and a PCR-free library was prepared using SMRTbell prep kit 3.0 (PacBio). Long-read WGS was done using a PacBio Revio system at an average coverage of approximately 30-fold. Putatively pathogenic differences between the wild-type sequence (human reference genome according to UCSC Genome Browser: hg19, GRCh37) and the patient’s sequence mentioned and interpreted in this report were assessed using an internally established quality system.

Identified variants were compared to literature and external as well as internal allele frequency databases (e.g., gnomAD). In addition, in silico analysis of the identified variants with regard to functional relevance, conservation, and splice effects was performed using bioinformatic prediction programs. The variants are classified using the current ACMG guidelines (23).

The genome data were filtered with respect to autosomal recessive, autosomal dominant, and X-linked mode of inheritance for very rare potentially pathogenic homozygous/putative compound heterozygous/heterozygous and hemizygous changes. For the filtered variants, a literature-based comparison using human mutation databases (e.g., HGMD, ClinVar) was performed according to the provided clinical information of the patient. In addition, the data were compared to public and internal allele frequency databases. Furthermore, the in silico scores of bioinformatic prediction programs were also taken into account. The NGS data were not analyzed for potentially pathogenic variants in genes not related to the requested indication. Additional, putatively relevant variants and carriership findings are not reported routinely.

### Short-read RNA-Seq of healthy blood samples.

For short read mRNA-Seq, 100 ng of DNase-treated total RNA (RIN > 8) was processed with the TruSeq Stranded mRNA Prep Kit (Illumina) including poly(A) selection. Indexes were added with the Illumina RNA UDI 384 v2 kit (IDT). Sequencing (150-bp paired-end) was performed with a NovaSeq X system (Illumina). Sequenced reads were aligned to the human reference genome (GRCh38) and were counted on the gene level with Rsubread (version 2.18) (https://bioconductor.org/packages/release/bioc/html/Rsubread.html). Differentially expressed genes (DEGs) were determined between control and treated samples employing edgeR (v4.2.1) (https://bioconductor.org/packages/release/bioc/html/Rsubread.html) and limma (v3.60.3) (https://bioconductor.org/packages/release/bioc/html/limma.html). Gene set enrichment analysis of DEGs was carried out with fgsea (v1.30.0) (https://bioconductor.org/packages/release/bioc/html/fgsea.html). Sashimi blots were generated with Integrative Genomics Viewer (IGV, version 2.17.4 03/26/2024) (https://igv.org/).

### Long-read RNA-Seq of healthy blood samples and organoids.

Using the Kinnex full-length RNA kit (Pacific Biosciences), poly(A)-RNA without prior fragmentation was reverse transcribed into cDNA, amplified, and concatenated to SMRT bell libraries with an approximately 16 kb insert size (MAS Seq method). Sequencing was performed on a Revio platform (Pacific Biosciences) at 24 hours video time.

HiFi circular consensus (CCS) reads were processed using the PacBio Iso-Seq pipeline (v4.3.0). Skera (v1.4.0) (https://anaconda.org/bioconda/pbskera) was used for deconcatenation. Primers were removed with lima (v2.13.0) (https://anaconda.org/bioconda/pbskera), and full-length reads were refined and clustered into high-confidence isoforms. Reads were aligned to the GRCh38 primary assembly using pbmm2 (v1.17.0) (https://anaconda.org/bioconda/pbmm2) with the ISOSEQ preset. Isoforms were collapsed using isoseq collapse and further classified and filtered with Pigeon (v1.4.0) (https://anaconda.org/bioconda/pbpigeon). For visualization, the refined reads were aligned. The GENCODE v45 genome and annotation were used throughout. Mapped long reads were visualized in IGV (version 2.17.4 03).

### Statistics.

All values are given as mean ± SEM. Comparisons between 2 groups were performed with unpaired Student’s *t* test. Analyses including more than 2 groups were performed with 1-way ANOVA followed by Dunnett’s multiple-comparison test. *P* values of less than 0.05 were considered significant. Statistical analyses were performed with Prism (GraphPad Software, v10.4.1).

### Study approval.

The individuals involved in this project presented at the Department of Ophthalmology at LMU Munich and research including patient samples were approved by the local ethics committee (ethics vote nr. 19-0226). Clinical research and publication of clinical imaging data was approved by the Independent Ethics Committee of Ludwig-Maximilians-University (ICE LMU), Munich (ethics vote no. 22-0897). Written informed consent for the use of patient samples and clinical imaging was received prior to participation and has been retained.

Human eyes, used for human retina analysis, were donated and collected in collaboration with the Eye Clinic Zurich and were approved by the local ethics committee (BASEC-Nr: PB_2020-01856).

All procedures with human samples and donations adhered to the tenets of the Declaration of Helsinki.

### Data availability.

Values for all data points shown in graphs are reported in the Supporting Data Values file. Sequencing data of human retinal organoids are accessible through NCBI GEO accession number GSE302995. Sequencing data of human samples have not been deposited in a public repository and are subject to controlled access to ensure the individuals’ privacy. Bioinformatics scripts/pipelines not mentioned in the Methods can be provided upon request.

## Author contributions

VJW, KSH, SM, and EB designed the research studies. VJW, MJG, AR, KSH, HJB, DYO, SVDE, and ZG conducted the experiments and acquired data. TH and VJW analyzed data. FB and IM provided human retina samples. HJB and CB organized and supervised diagnostics of P2, P4, P5, and P6, and PacBio long-read sequencing experiments. VJW, MJG, TH, and EB wrote the manuscript. EB, SM, and MB acquired funding. SM and MJG supervised experiments with human patient samples. EB supervised the project. All authors contributed to the final manuscript.

## Funding support

- Helmut-Ecker Stiftung (to EB).
- Novartis Pharma GmbH (to SM).
- Deutsche Forschungsgemeinschaft (325871075 to SM).
- Iten-Kohaut Stiftung (to EB).
- Swiss National Science Foundation (320030E_221942 to EB)
- Munich Medical & Clinician Scientist Program, LMU Munich (to MJG).

## Supplementary Material

Supplemental data

Unedited blot and gel images

Supplemental table 6

Supporting data values

## Figures and Tables

**Table 1:**
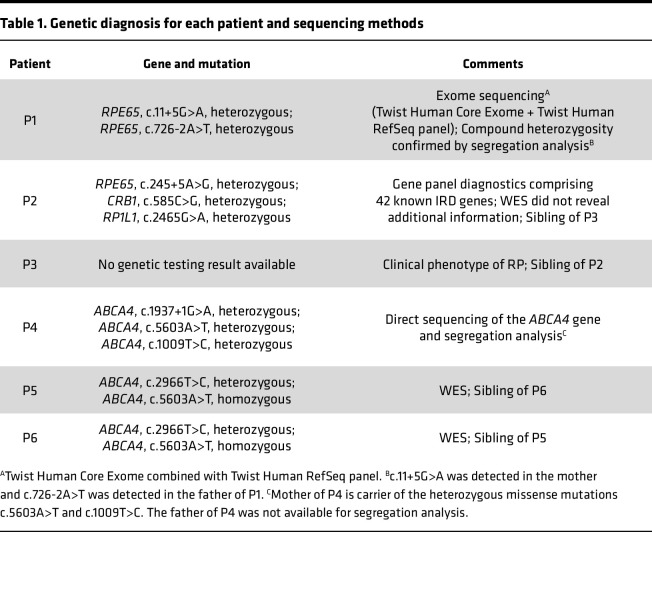
Genetic diagnosis for each patient and sequencing methods

## Version 1 10/07/2025

In-Press Preview

## Version 2 11/24/2025

Electronic publication
