# Supplementary material for: CRISPR/Cas-mediated activation of genes associated with inherited retinal dystrophies in human cells for diagnostic purposes
Source: JCI Insight. 2025 Sep 30;10(22):e189615. doi: 10.1172/jci.insight.189615 (PMC12643484; doi:10.1172/jci.insight.189615)
Supplement: Supplemental data [file jciinsight-10-189615-s233.pdf]

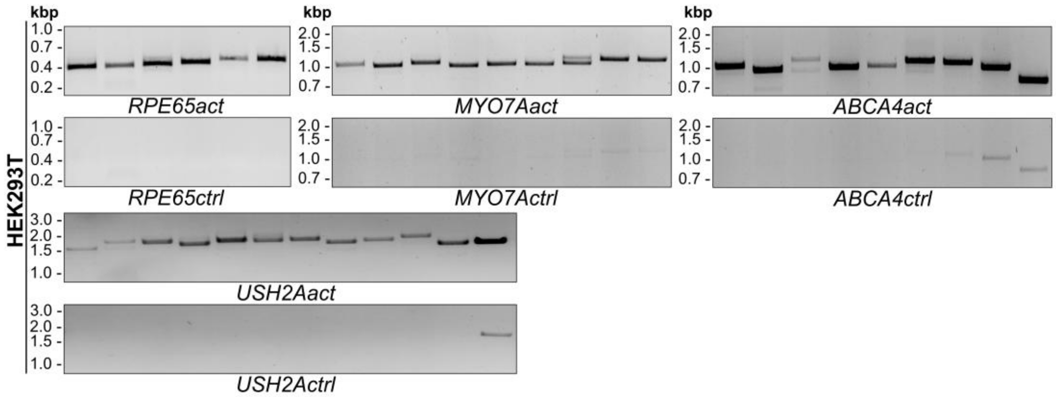

Supplemental Figure 1: Extended RT-PCR analyses of HEK293T containing control samples without transcriptional activation (related to Figure 1). For further details, see main text and Figure 1.

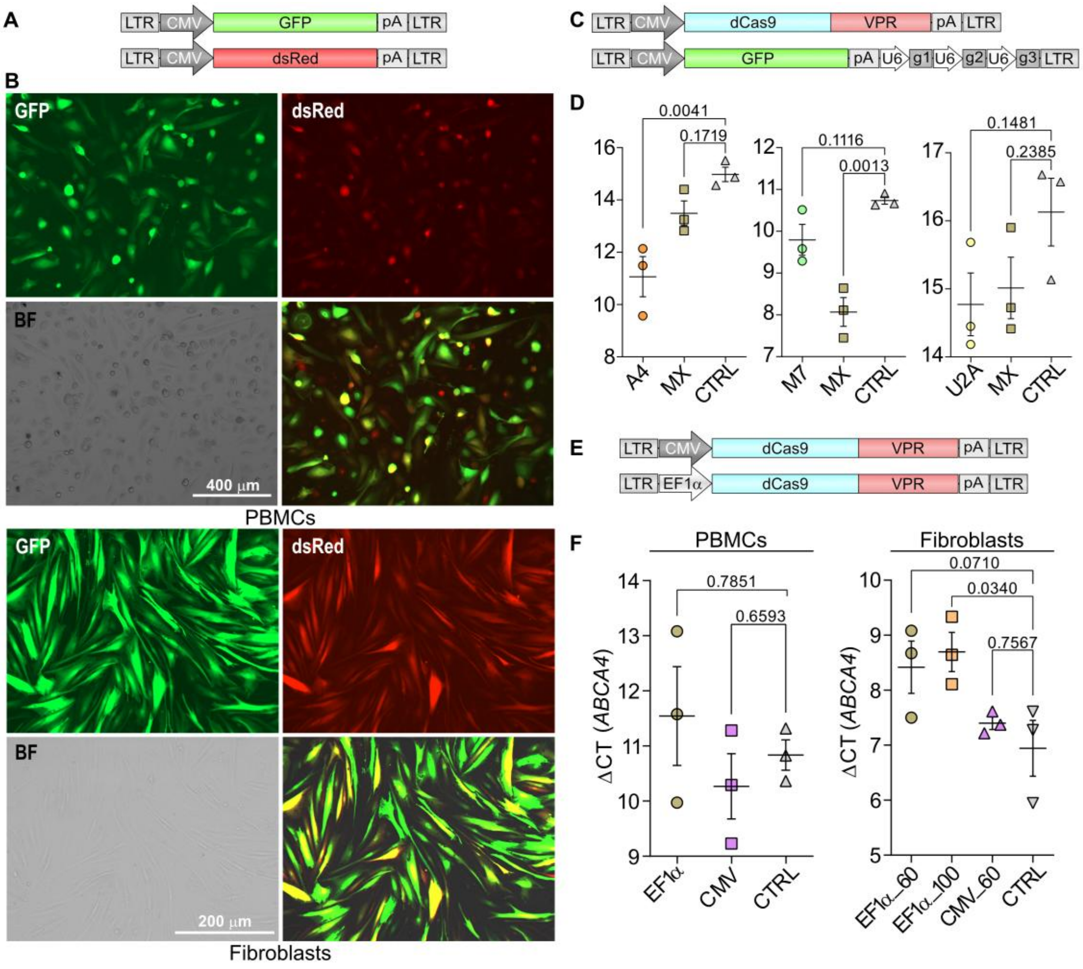

Supplemental Figure 2: Lentiviral transduction and optimization of CRISPRa in human PBMCs and fibroblasts. (A) Scheme depicting the LV vector expression cassettes used for evaluation of co-transduction. GFP, green fluorescent protein. LTR, long terminal repeats. **(B)** Microscopic imaging of human PBMCs and fibroblasts co-transduced with LVs expressing either GFP or dsRed. For this experiment, PBMCs have been cultured for two and fibroblasts for one week before transduction. BF, bright field. **(C)** Scheme showing lentiviral expression plasmids for dCas9-VPR mediated gene activation. **(D)** RT-qPCR showing target gene expression after dual transduction of HEK293T (n = 3). **(E)** Scheme depicting LV expression cassettes for promoter evaluation. EF1α, Elongation factor 1α promoter. **(F)** RT-qPCR showing *ABCA4* expression after transduction of PBMCs with LVs expressing dCas9-VPR under control of CMV or EF1α promoter (left; n = 3) and with different multiplicities of infections (right; n = 3). Values are given as mean ± SEM. Statistics/Multiple comparisons were performed with one-way ANOVA and Dunnett's test.

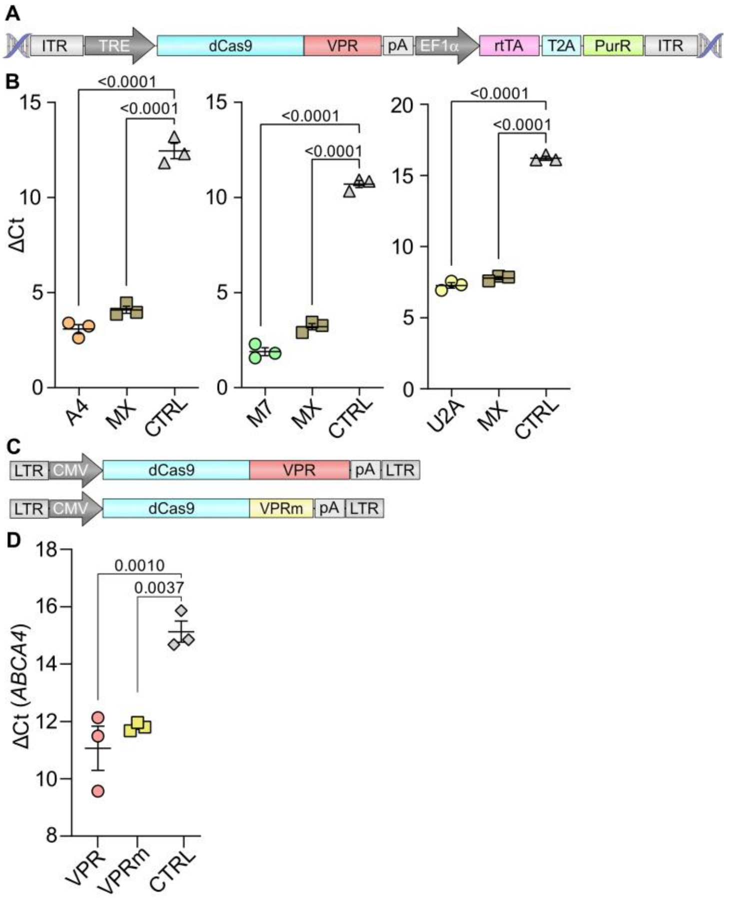

Supplemental Figure 3: Generation of HEK293T cells stably expressing dCas9-VPR and evaluation of a smaller CRISPRa module. (A) PiggyBac gene expression vector used for integration in the HEK293T genome. ITR, inverted terminal repeats; TRE, tetracycline responsive element; rtTA, reverse tetracycline-controlled transactivator. **(B)** RT-qPCR result for each gene activation (n = 3). A4, *ABCA4*; M7, *MYO7A*; U2A, *USH2A*; MX, multiplexing with all three sgRNA cassettes. **(C)** Plasmid scheme for LVs expressing dCas9 in combination with VPR or VPRmini (VPRm). **(D)** RT-qPCR result for *ABCA4* expression after co-transduction of HEK293T (n = 3). All values are shown as mean ± SEM. Statistics/Multiple comparisons were performed with one-way ANOVA and Dunnett's test.

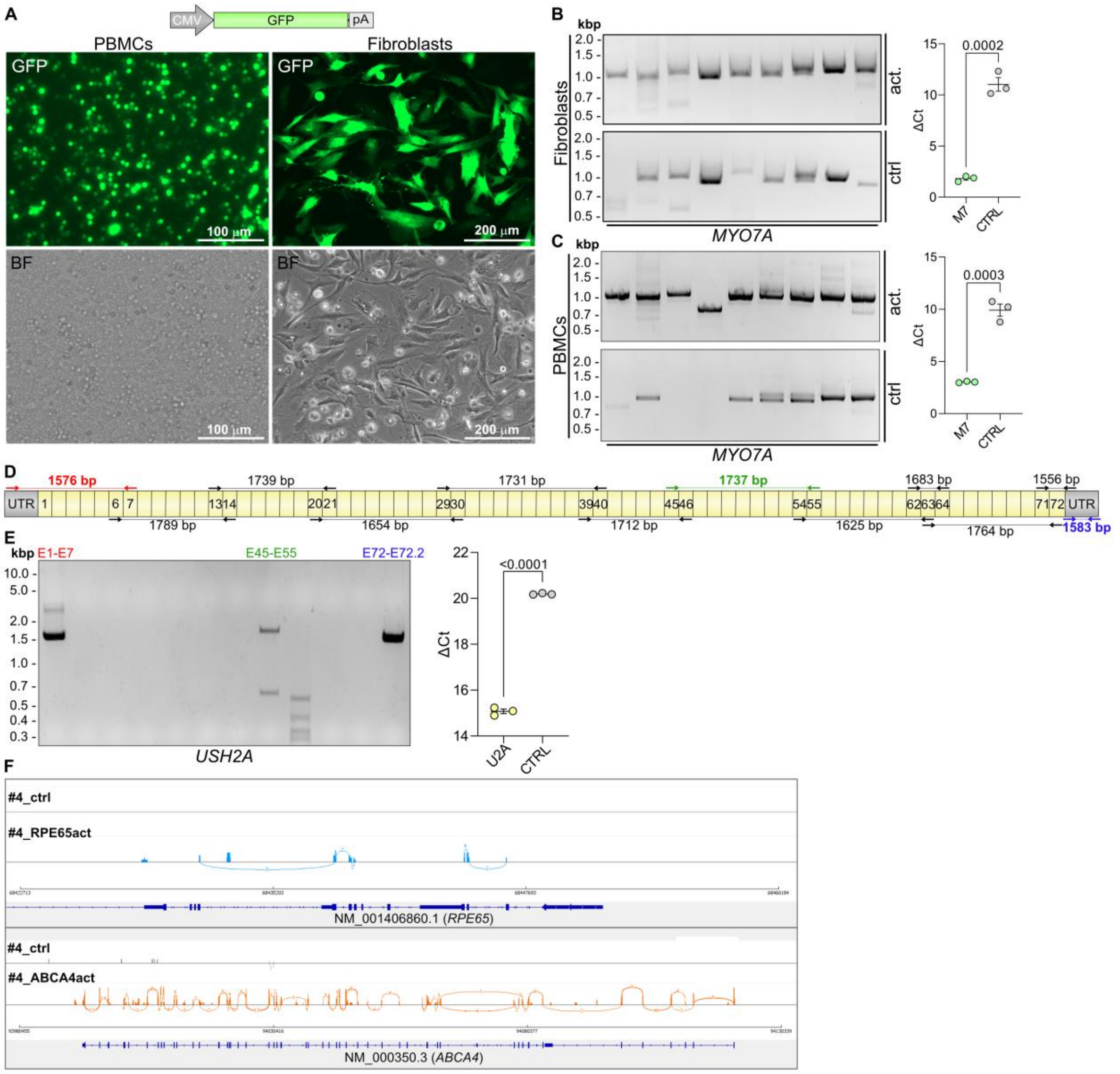

Supplemental Figure 4: Nucleofection of primary target cells and evaluation of transcriptionally activated *MYO7A* and *USH2A*. (A) Upper panel: GFP reporter plasmid used for nucleofection. Lower panel: Live imaging of PBMCs and fibroblasts after nucleofection with the GFP reporter plasmid. **(B)** RT-PCR (left) and RT-qPCR (right; n = 3) for *MYO7A* in fibroblasts after nucleofection and transcriptional activation. **(C)** RT-PCR (left) and RT-qPCR (right) for *MYO7A* in PBMCs after nucleofection and transcriptional activation. **(D)** Primer design for the RT-PCR analysis of the *USH2A* transcript. **(E)** *USH2A* amplifications (left) of exon 1-7 (E1-E7, red), exon 45-55 (E45-E55, green) and exon 72-72.2 (E72-E72.2, blue) as indicated in D. RT-qPCR (right) for *USH2A* after nucleofection of PBMCs with CRISPRa plasmids (n = 3). **(F)** Sashimi blot for transcriptionally activated *RPE65* (blue) and *ABCA4* (orange) compared to untreated control PBMCs (grey). Blue and orange bars represent the respective exon coverage. The numbered curved lines show the number of reads spanning of exon-exon junctions. Dark blue schemes highlight the main reference transcript of the respective genes. All values are shown as mean ± SEM. Statistics are calculated with Student's t-test.

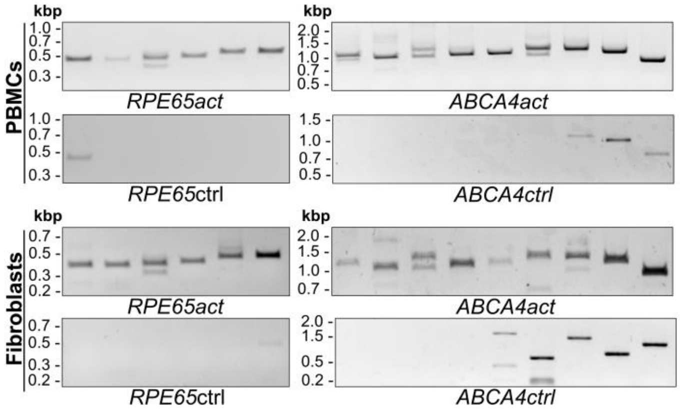

Supplemental Figure 5: Extended RT-PCR analyses of PBMCs and fibroblasts containing control samples without transcriptional activation (related to Figure 2). For further details, see main text and Figure 2.

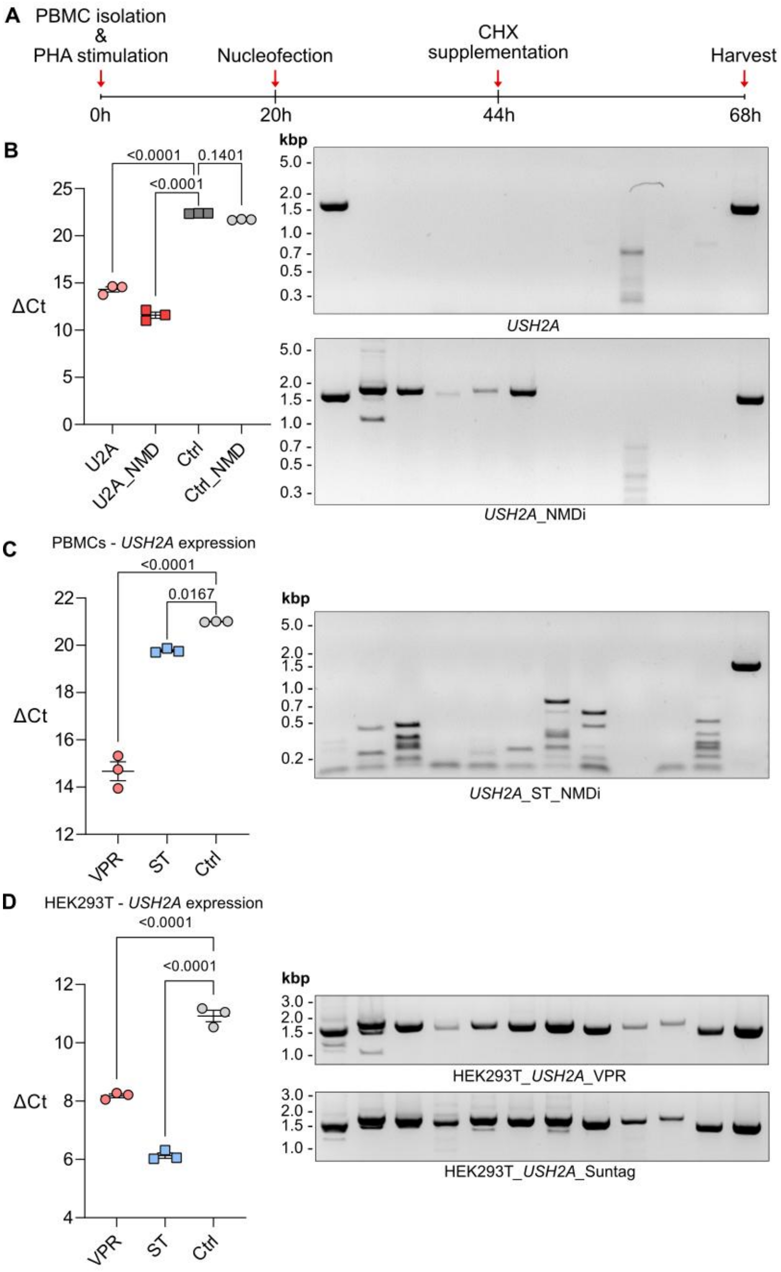

Supplemental Figure 6: Evaluation of NMD inhibition and alternative transactivation domains for activation of *USH2A*. (A) Experimental design for the CHX-mediated NMD inhibition. CHX, Cycloheximide. NMD, Nonsense-mediated mRNA decay. **(B)** RT-qPCR (left; n = 3) and RT-PCR (right) for *USH2A* expression analysis after NMD inhibition in PBMCs in comparison to *USH2A* expression without NMD inhibition and naive control samples. **(C)** RT-qPCR (left; n = 3) and RT-PCR (right) after p65-HSF1/Suntag-mediated transcriptional activation (ST) in comparison to VPR-mediated transcriptional activation and naive controls. **(D)** RT-qPCR (left; n = 3) and RT-PCR (right) to compare the activation efficiency of *USH2A* between VPR and p65-HSF1/Suntag (ST) in HEK293T. All values are shown as mean ± SEM. Statistics/Multiple comparisons were performed with one-way ANOVA and Dunnett's test.

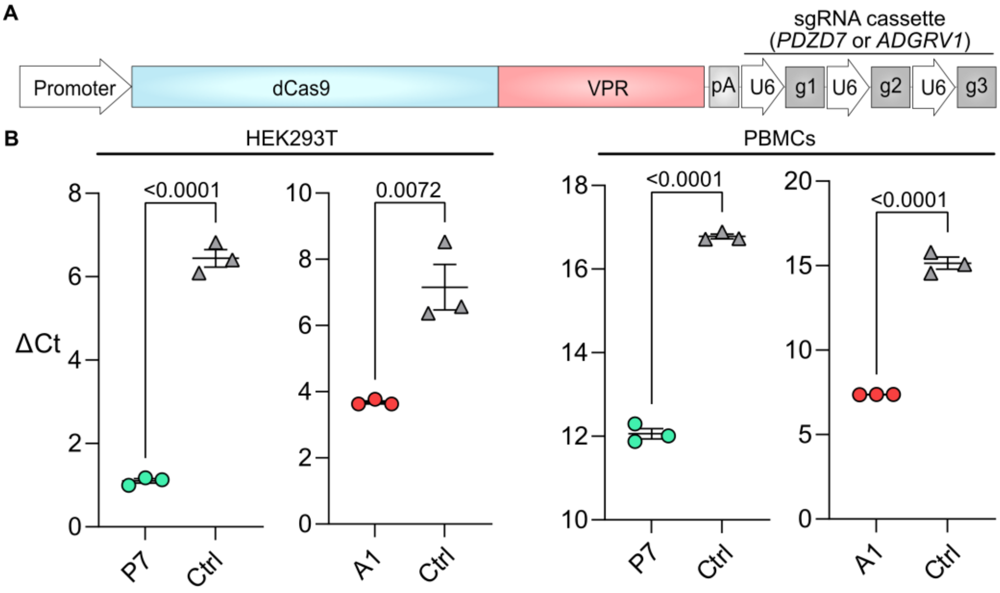

Supplemental Figure 7: Transcriptional activation of *PDZD7* and *ADGRV1* in HEK293T and PBMCs. (A) dCas9-VPR expression vector with sgRNA cassette targeting either *PDZD7* or *ADGRV1*. **(B)** RT-qPCR after transcriptional activation of *PDZD7* (P7) and *ADGRV1* (A1) in HEK293T (left) and PBMCs (right) in comparison to naive controls (n = 3). All values are shown as mean ± SEM. Statistics were done using the Student's t-test.

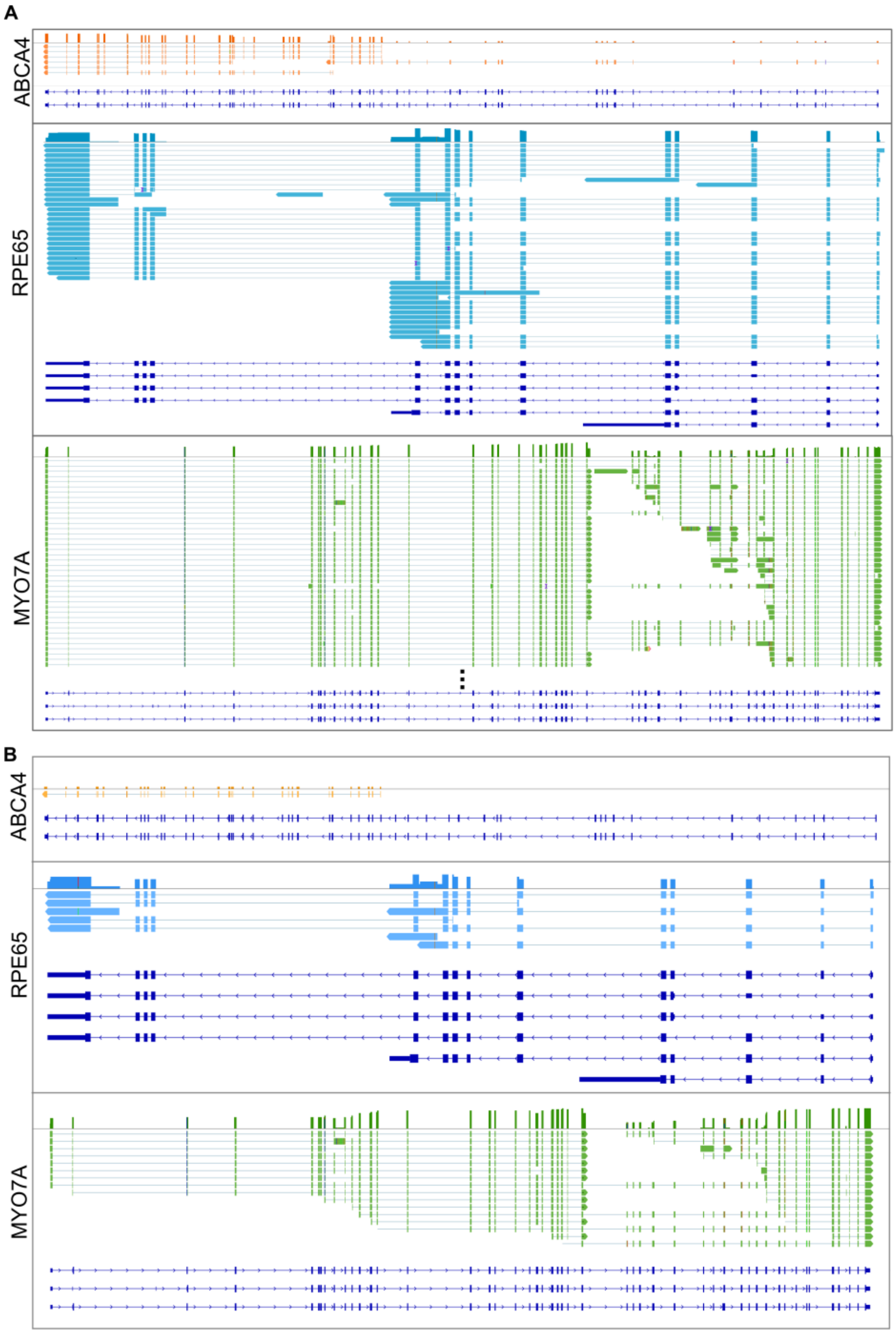

Supplemental Figure 8: Consensus sequences for *ABCA4*, *RPE65* and *MYO7A* after long-read sequencing. (A) Sequencing reads for *ABCA4* (orange), *RPE65* (blue) and *MYO7A* (green) after transcriptional activation in human PBMCs. The colored bars on top of each consensus sequence panel represents the exon coverage. Detected mutations are labelled in red, bright green, orange or blue. Dark blue schemes highlight the reference transcripts of the respective genes. **(B)** Consensus sequences for *ABCA4* (orange), *RPE65* (blue) and *MYO7A* (green) after transcriptional activation in human PBMCs.

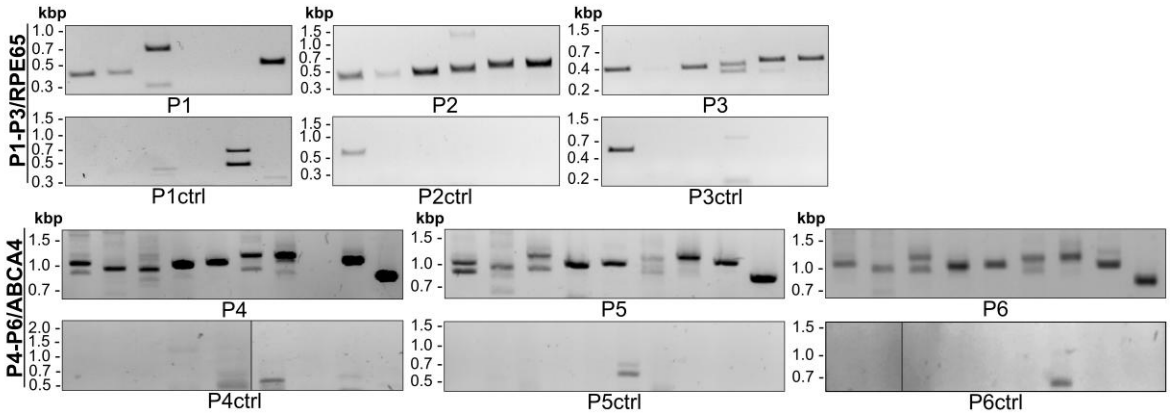

Supplemental Figure 9: Extended RT-PCR analyses of patient samples containing control samples without transcriptional activation (related to Figure 5). For further details, see main text and Figure 5.

Supplemental Table 1: Sanger sequencing result of *ABCA4* amplicons

Exon range	Band identity
E1 – 8	Upper: Canonical sequence (Exon 1-8)<br>Lower: Exon 3 skipping (PTC)
E7 – 10	Constitutive splicing
E11 – 16	Upper: Canonical sequence (Exon 11-16)<br>Lower: Exon 15 skipping (in frame)
E15 – 21	Constitutive splicing
E20 – 27	Constitutive splicing
E26 – 35	Upper: Canonical sequence (Exon 26-35)<br>Lower: Exon 28+29 skipping (PTC)
E34 – 42	Constitutive splicing
E41 – 48	Constitutive splicing
E47 – 50	Constitutive splicing

Supplemental Table 2: Sanger sequencing result of *RPE65* amplicons

Exon range	Band identity
E1 – 4	Constitutive splicing I
E3 – 6	Constitutive splicing
E5 – 8	Upper: Canonical sequence (Exon 5 - 8)<br><br>Lower: Exon 7 skipping (PTC)
E7 – 10	Constitutive splicing
E9 – 13	Constitutive splicing
E12 – 14	Constitutive splicing

Supplemental Table 3: sgRNA sequences

sgRNA	Sequence [5' -> 3']
sgRPE65_1	CCCAAATAAAGCCAAGCATC
sgRPE65_2	GGGGTGACTGGGATCAGCTC
sgRPE65_3	CCTCCCTCAGCTGAAGGGGT
sgABCA4_1	TTACACATAAGCCCTTAGTT
sgABCA4_2	TACCTAAAGACATCCCCCTC
sgABCA4_3	CCCTAAAGACACAAGTGGCT
sgMYO7A_1	GGCGGTGTCCTGCAAACAAC
sgMYO7A_2	CAGAGCACTGGCTGCCGACA
sgMYO7A_3	GGGTGCGGTGACATCAGCGT
sgUSH2A_1	TCTATCAATGGGCCAAGCTT
sgUSH2A_2	AGAGGGGGGATTATAGGATA
sgUSH2A_3	GAGGTACCAGCGGAAAGCTT
sgADGRV_1	ACTCGCGCGCTCCTAAGCCG
sgADGRV_2	GTGTTCCCGACGCCAGAGCG
sgADGRV_3	TCACCTGACCAGCGGAGCGG
sgPDZD7_1	AGGGACAGCCGAGCGTTACC
sgPDZD7_2	AAGAGGTGGTTCGAGCCGGA
sgPDZD7_3	CTGAGCCGAGCCAGCTACCG

Supplemental Table 4: RT-PCR primer for *RPE65*, *ABCA4*, *MYO7A* and *USH2A* transcript amplification

Primer	Sequence [5' -> 3']
RPE65_E1_fw	GCAGTTGGTGCCAGAACTC
RPE65_E3_fw	CACCTGTTTGATGGGCAAGC
RPE65_E5_fw	ACGCTTGCACAGAGACCAAC
RPE65_E7_fw	TACAATTCCCCTGCAGTGACC
RPE65_E9_fw	TGTGGATCTCTGCTGCTGGA
RPE65_E12_fw	ATGCGTATGGACTTGGCTTGA
RPE65_E4_rev	TTGCAGGGATCTGGGAAAGC
RPE65_E6_rev	TTTCAATGTGGGGGTGAGCA
RPE65_E8_rev	GTAGTTGGCTCCCCAAAGACT
RPE65_E10_rev	ACTTCAGGTTGGGGAGCCTT
RPE65_E13_rev	CTTCCAAGGCATCTGGGTGA
RPE65_E14_rev	TGCTTGCTCAACTCAGTGCT
ABCA4_E1_fw	GAGCCAGAGGCGCTCTTAAC
ABCA4_E7_fw	TCCCACACTCCTAGACAGCC
ABCA4_E11_fw	ATACCCTGGGGAACCCAAC
ABCA4_E15_fw	TTACAGCGACCCATTCATCCTC
ABCA4_E20_fw	GTCCATCCTGACGGGTCTG
ABCA4_E26_fw	CTGAAGGTCACGGAGGATTCTG
ABCA4_E34_fw	GGCCCTATCACTAGAGAGGC
ABCA4_E41_fw	CCACTAAGGAGCCCATTGTTGAT
ABCA4_E47_fw	CTGGCCATCATGGTAAAGGGC
ABCA4_E8_rev	CTCTGGACCACCATTCTGCAT
ABCA4_E12_rev	CTTCACGTGGGGTGGTAGAG

Primer	Sequence [5’ -> 3’]
ABCA4_E16_rev	GGACTGTTCCCGATGTTGCT
ABCA4_E21_rev	TCCAACATGGCTTCCATCTCC
ABCA4_E27_rev	GTCGGGGGTTGACGTTTTC
ABCA4_E35_rev	CCGTAAGATGGCGTTGTGGG
ABCA4_E42_rev	GCTGGAGGTGCCTGGATAAA
ABCA4_E48_rev	CAGTGTGGTCTGTGTGACTGA
ABCA4_E50_rev	TCCATGAAAATCACACACAACGCA
MYO7A_E1_fw	CTGGACAGCTGCTCTGGG
MYO7A_E6_fw	GGGAAGACGGAGAGCACAAA
MYO7A_E12_fw	GTGGACAAGATCAACGCAGC
MYO7A_E18_fw	CGATGACTGGCAGATAGGCAA
MYO7A_E23_fw	GAGCTGAAGGAGAAGGAGGC
MYO7A_E28_fw	TTCTCGTGTCTCTCTGCGTG
MYO7A_E33_fw	CCAGAGGAGAACTGATGCCC
MYO7A_E38_fw	ACCCAAGCACACGCTGAG
MYO7A_E45_fw	GTATCTCCGAGGCTACCACAAG
MYO7A_E7_rev	ACGTGACTTTTCCAGCAGGT
MYO7A_E13_rev	TCGATGTGCAGCCAGTCAAT
MYO7A_E19_rev	ATGACTCTGTCGGTGATGGC
MYO7A_E24_rev	TCACCCTCGTCGTCATGGTA
MYO7A_E29_rev	ACAAAGGTCCTTCTCAGGCG
MYO7A_E34_rev	GGCCATGATCTCTGGGAAGG
MYO7A_E39_rev	CTGCACATATGCCTCGTCCT
MYO7A_E46_rev	CGTGCTTGTTGAAGTAGGCG
MYO7A_E49_rev	GATCAGGGTAGACTTGAGCAGG
USH2A_E1_fw	TGTTTGCTCTGCAGAATACTT

Primer	Sequence [5’ -> 3’]
USH2A_E6_fw	GTTGGTACTTCATGGGTTTCA
USH2A_E13_fw	GATGTGTGAGTGTGATTCCTT
USH2A_E20_fw	CCCAAGAACTATCTTACACTG
USH2A_E29_fw	AGTCGAGGACGTACAACAG
USH2A_E39_fw	GATGAGGTCTGGCGACT
USH2A_E45_fw	CAGGACTTCATGCAACCA
USH2A_E54_fw	TATCAGCTGAAAGCTTGCACG
USH2A_E62_fw	GTAGAACAGAAAGAGAATGGC
USH2A_E63_fw	ATAGTAAACCAGCTGAAGCC
USH2A_E71_fw	CTCATGGACATTCAAGACAAG
USH2A_E72_fw	CTCTGTCTAACACACAATAGT
USH2A_E7_rev	TTTCCGTTGGTTGTGGACTA
USH2A_E14_rev	TGGCATTGCCTGGAGAAATA
USH2A_E21_rev	TTCCTTTAACCAGAGGTGGC
USH2A_E30_rev	CAGGTCACCTCAATGCTGTAT
USH2A_E40_rev	ACTATGTGCACTGCCAAATCC
USH2A_E46_rev	GAGATGTCCAGATGACACGTA
USH2A_E55_rev	CTTGGGTAGTAGCTGCAACTA
USH2A_E63_rev	CCGTCACTGAAGATGTTGTAT
USH2A_E64_rev	CATCAAAGGTGCAATCTCAG
USH2A_E72_rev	GTTCATCAGGTCCTCTTCAT
USH2A_E72.1_rev	GACTATCCCTTACTTTACAAG
USH2A_E72.2_rev	GGTCACATTCAAATATTTATTAA

Supplemental Table 5: RT-qPCR primer

Primer	Sequence [5’ -> 3’]
qPCR_RPE65_fw	CAGCTCATGTAACAGGCAGGA
qPCR_RPE65_rev	GCTTGCCCATCAAACAGGTG
qPCR_ABCA4_fw	GGCCTCCACCACAAGCGGAATG
qPCR_ABCA4_rev	CAGGAGCAGATCCCAGATTGAGCGT
qPCR_MYO7A_fw	TGGTGATTCTTCAGCAGGGG
qPCR_MYO7A_rev	GGAGAGATCCAGTGTTCATTGTC
qPCR_USH2A_fw	CAGGCCAGTGCAAGTGCAAAGC
qPCR_USH2A_rev	ACTGGCAGGGCTCACATCCAAC
qPCR_ADGRV1_fw	ACTTGGAAGGAGGAGTAGCTGA
qPCR_ADGRV1_rev	TTGGGAAGCCACTCCTAGACT
qPCR_PDZD7_fw	CTCCAAGACGCTGATGAACCT
qPCR_PDZD7_rev	ACGCCCCCTGCTTTCTCT
