# Supplementary material for: CRISPR/Cas-mediated activation of genes associated with inherited retinal dystrophies in human cells for diagnostic purposes
Source: JCI Insight. 2025 Sep 30;10(22):e189615. doi: 10.1172/jci.insight.189615 (PMC12643484; doi:10.1172/jci.insight.189615)

Full unedited gel for Figure 1C and S.Figure 1 (ABCA4)

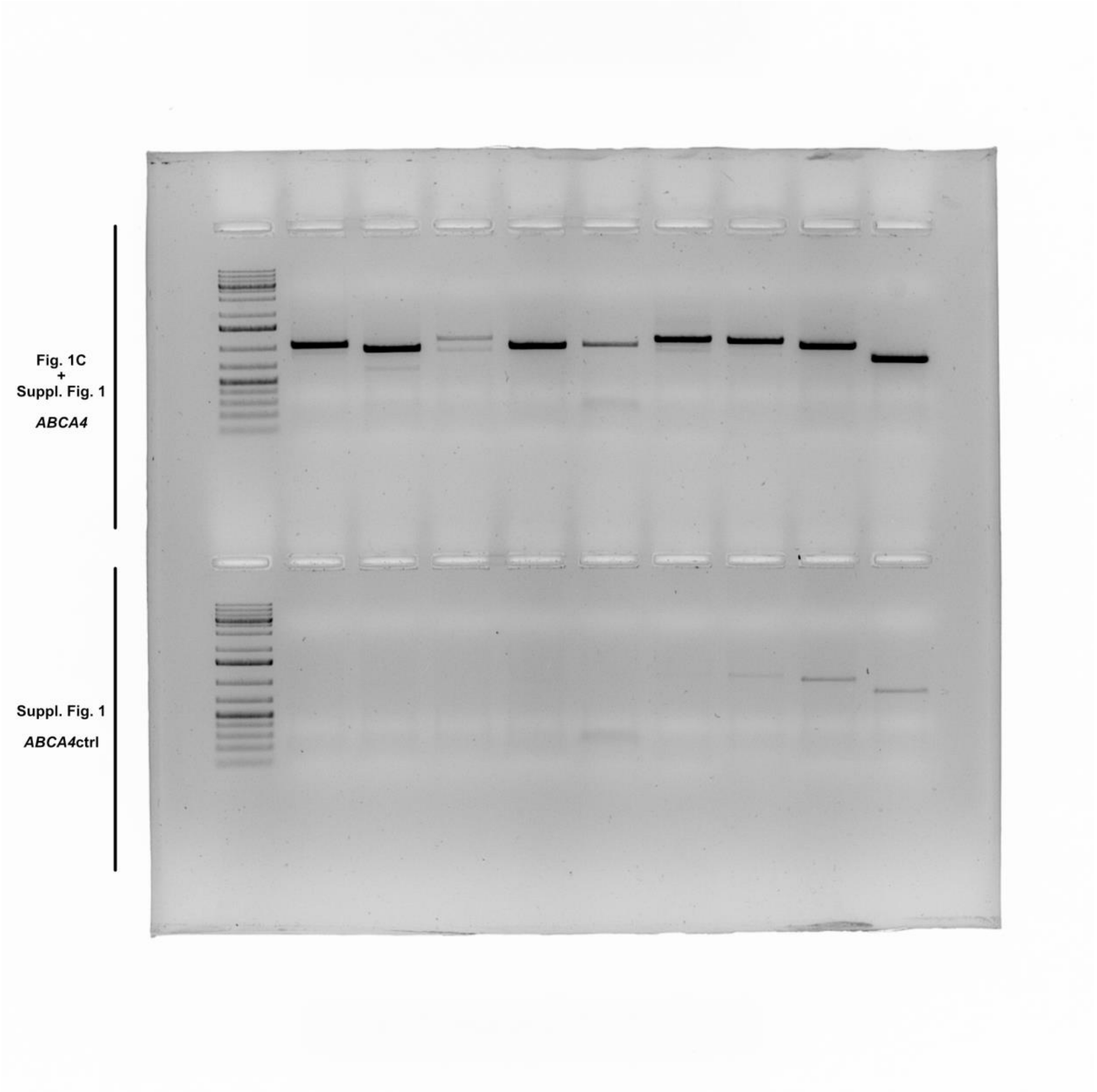

Full unedited gel for Figure 1C and S.Figure 1 (MYO7A)

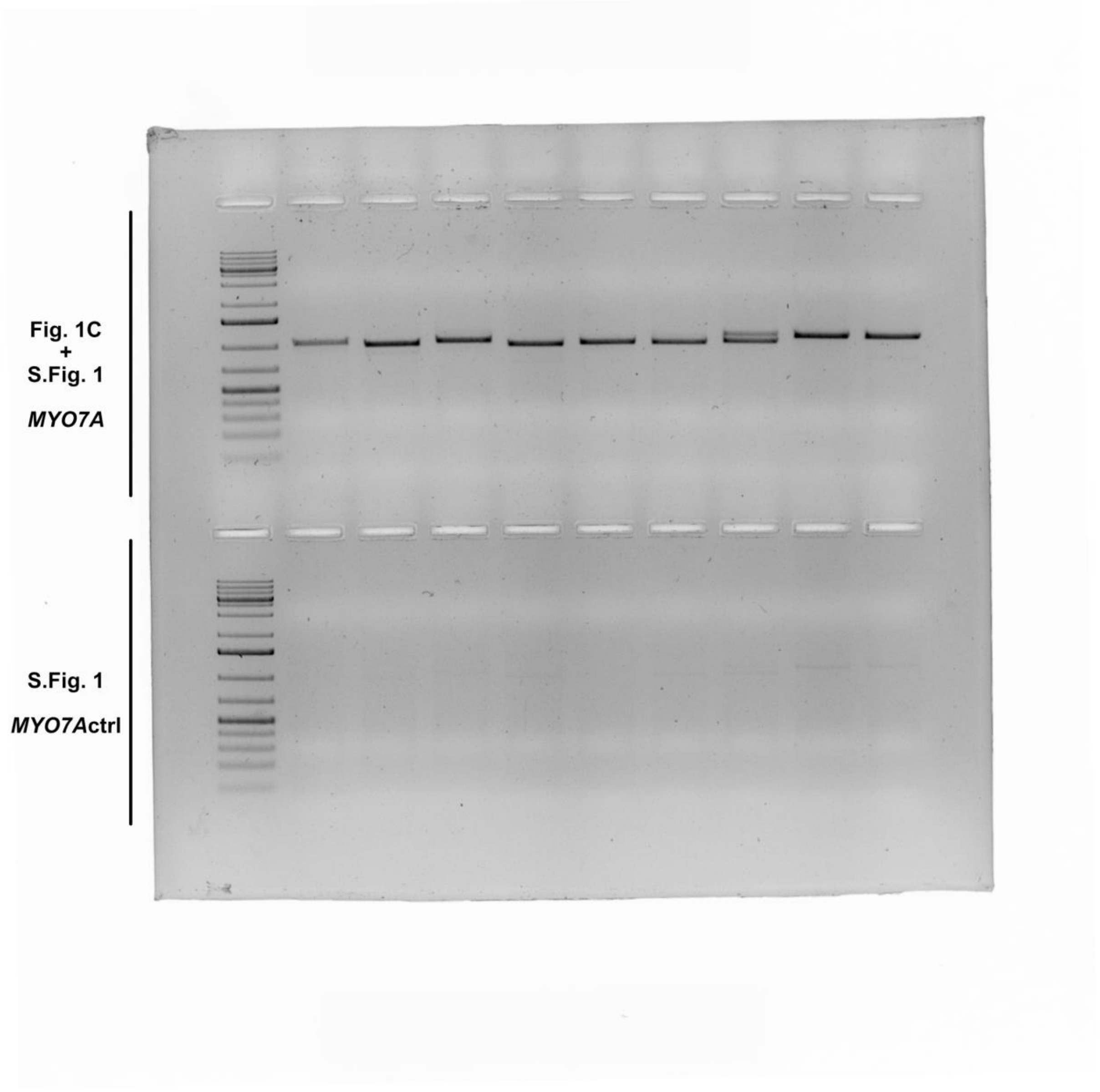

Full unedited gel for Figure 1C and S.Figure 1 (RPE65)

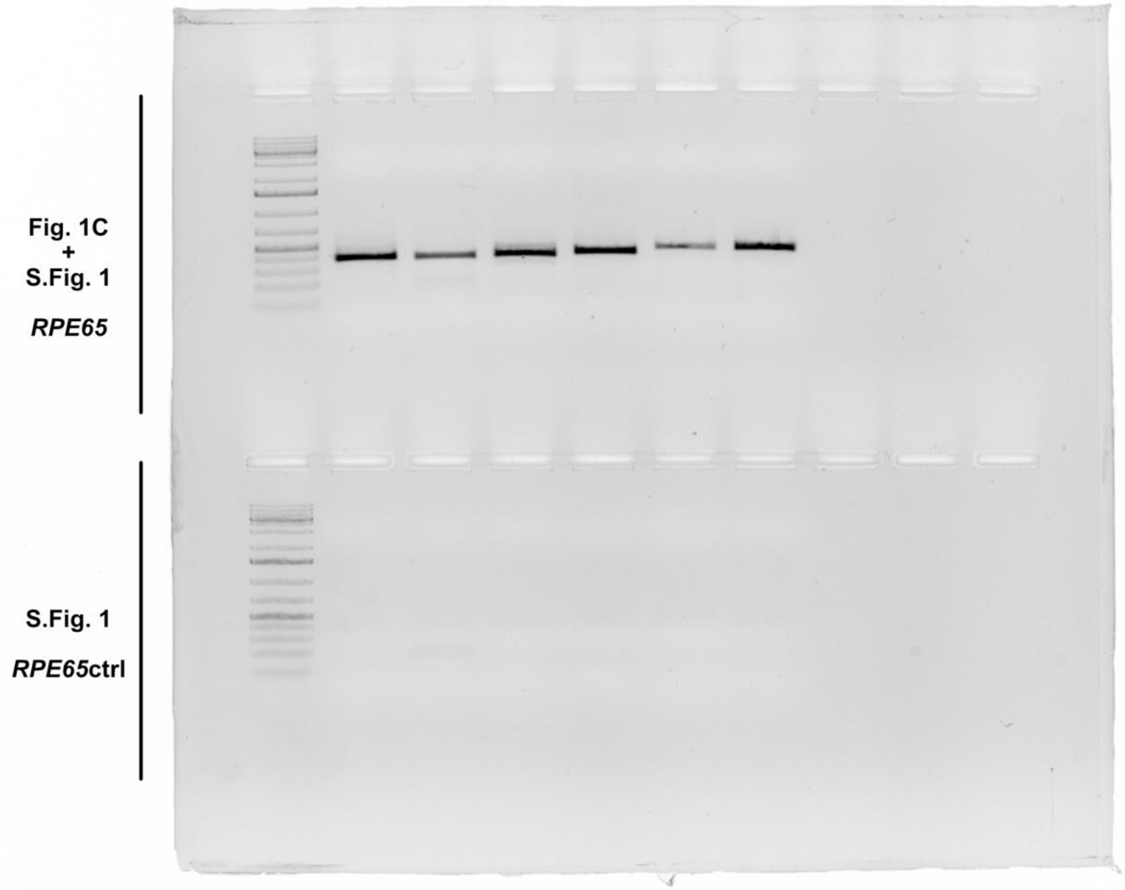

Full unedited gel for Figure 1C and S.Figure 1 (USH2A)

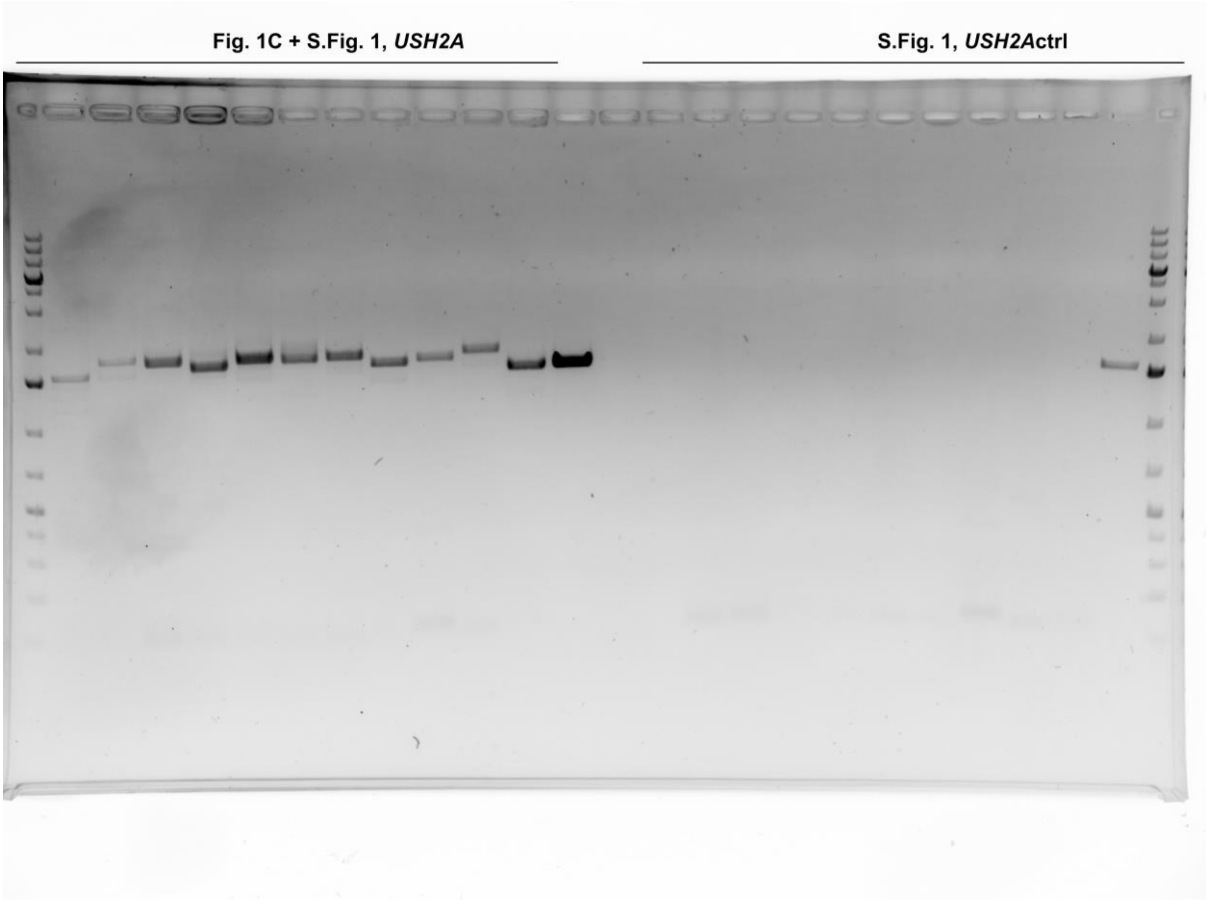

Full unedited gel for Figure 2A (ABCA4,hRE)

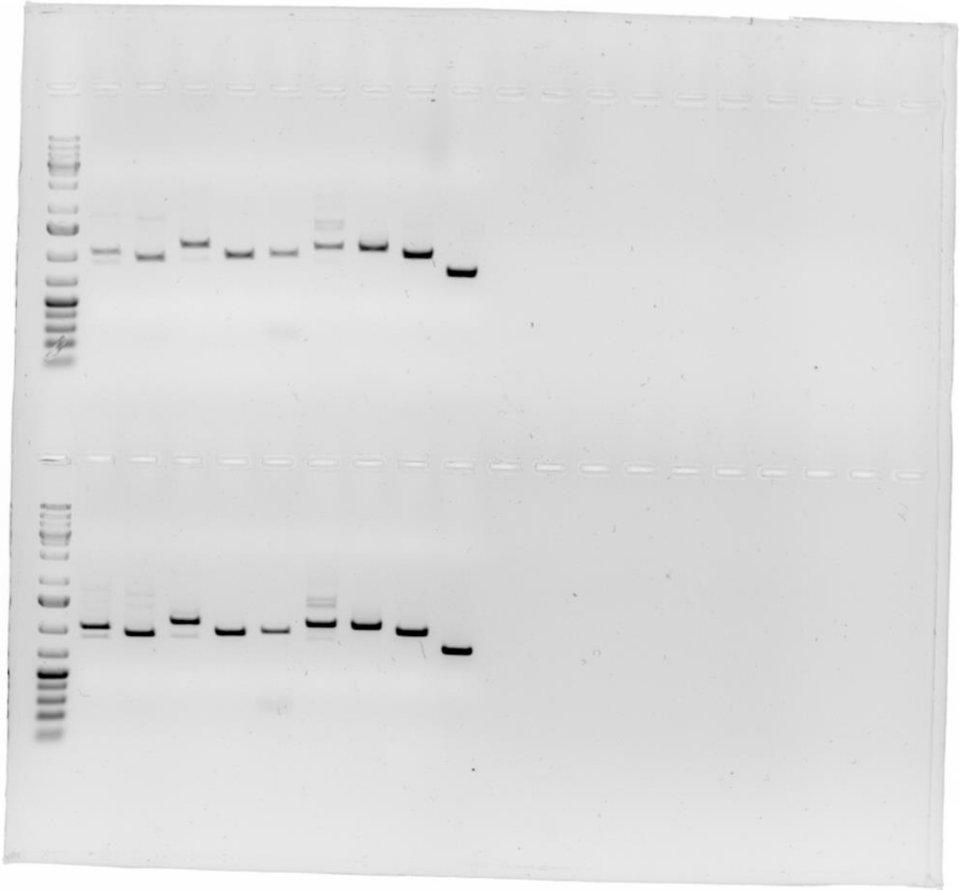

Fig.2A

ABCA4, hRE

Full unedited gel for Figure 2A (ABCA4,hRO)

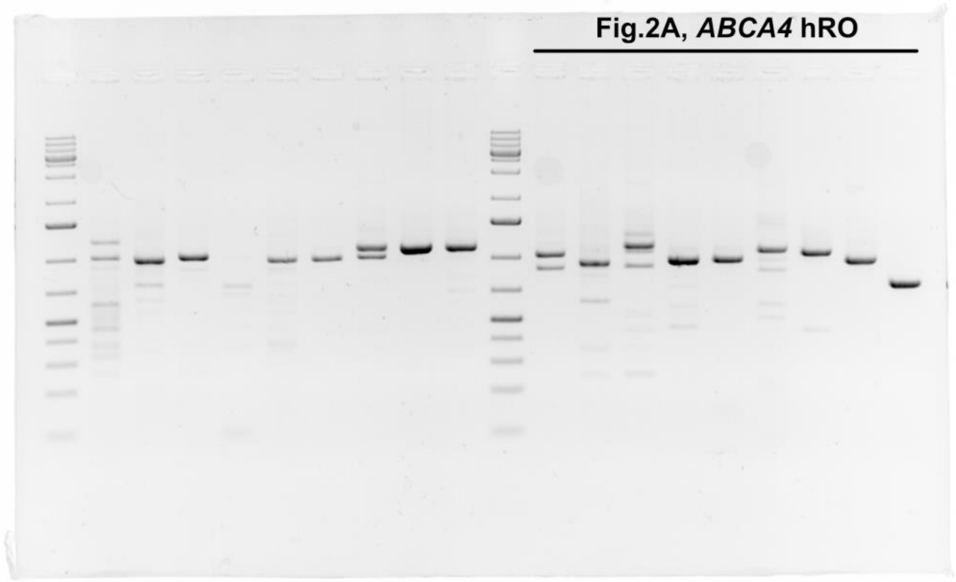

Full unedited gel for Figure 2A (RPE65,hRE)

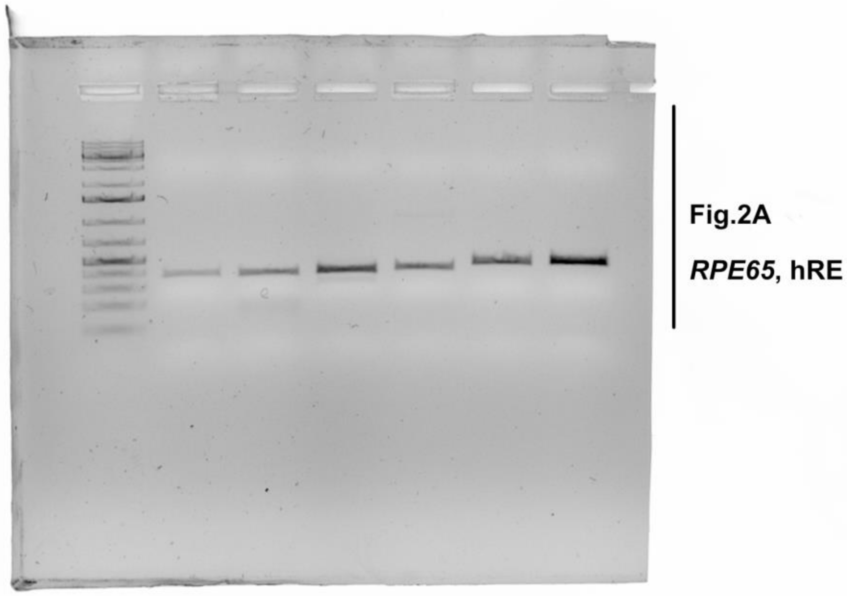

Full unedited gel for Figure 2A (RPE65,hRO)

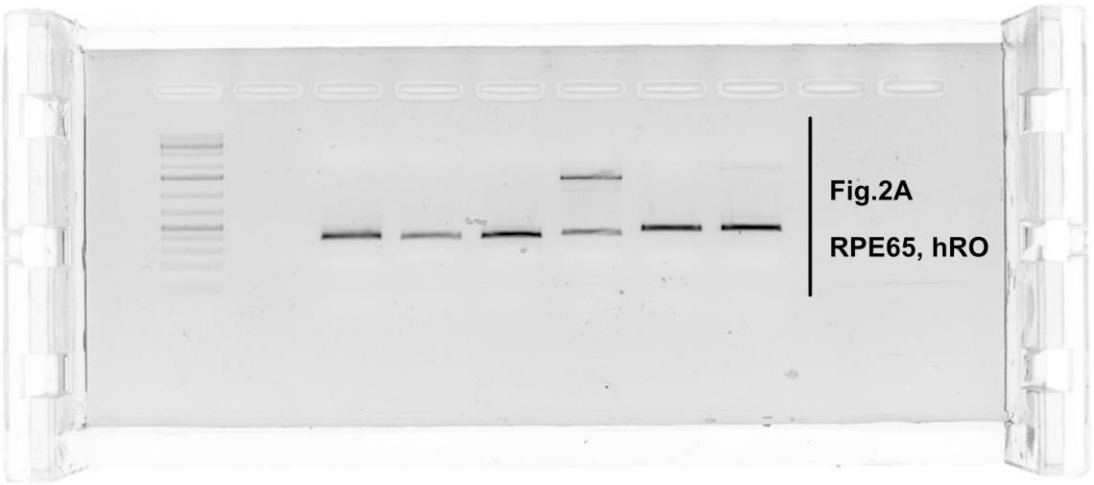

Full unedited gel for Figure 2A and S.Figure 5 (ABCA4,Fibroblast)

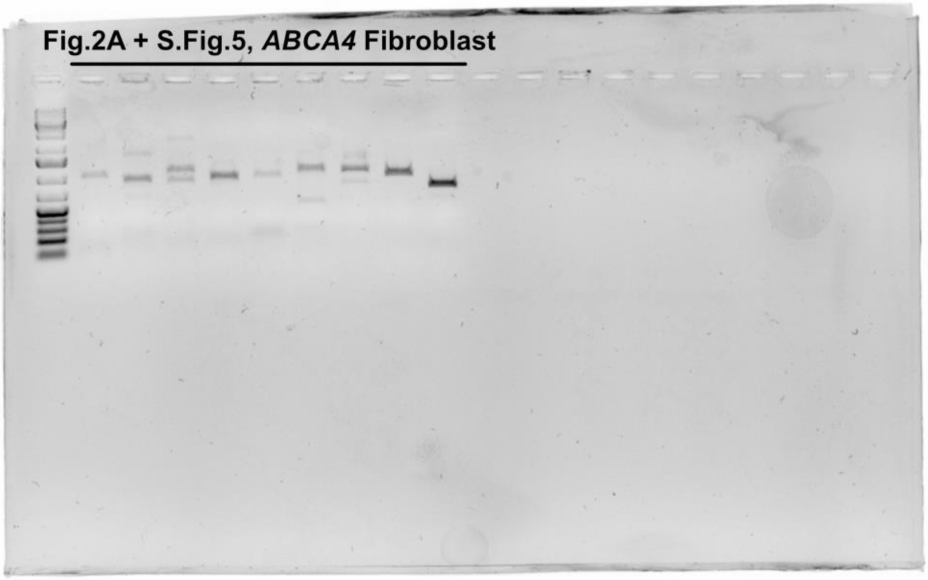

Full unedited gel for Figure 2A and S.Figure 5 (ABCA4,PBMC)

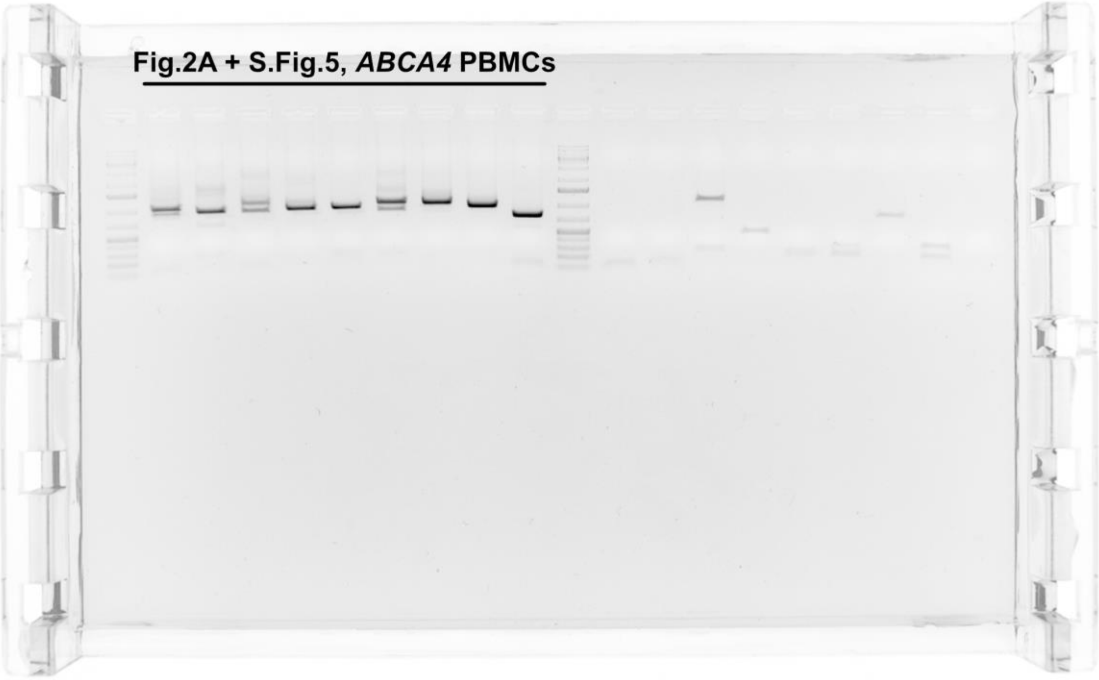

Full unedited gel for Figure 2A and S.Figure 5 (RPE65,Fibroblast)

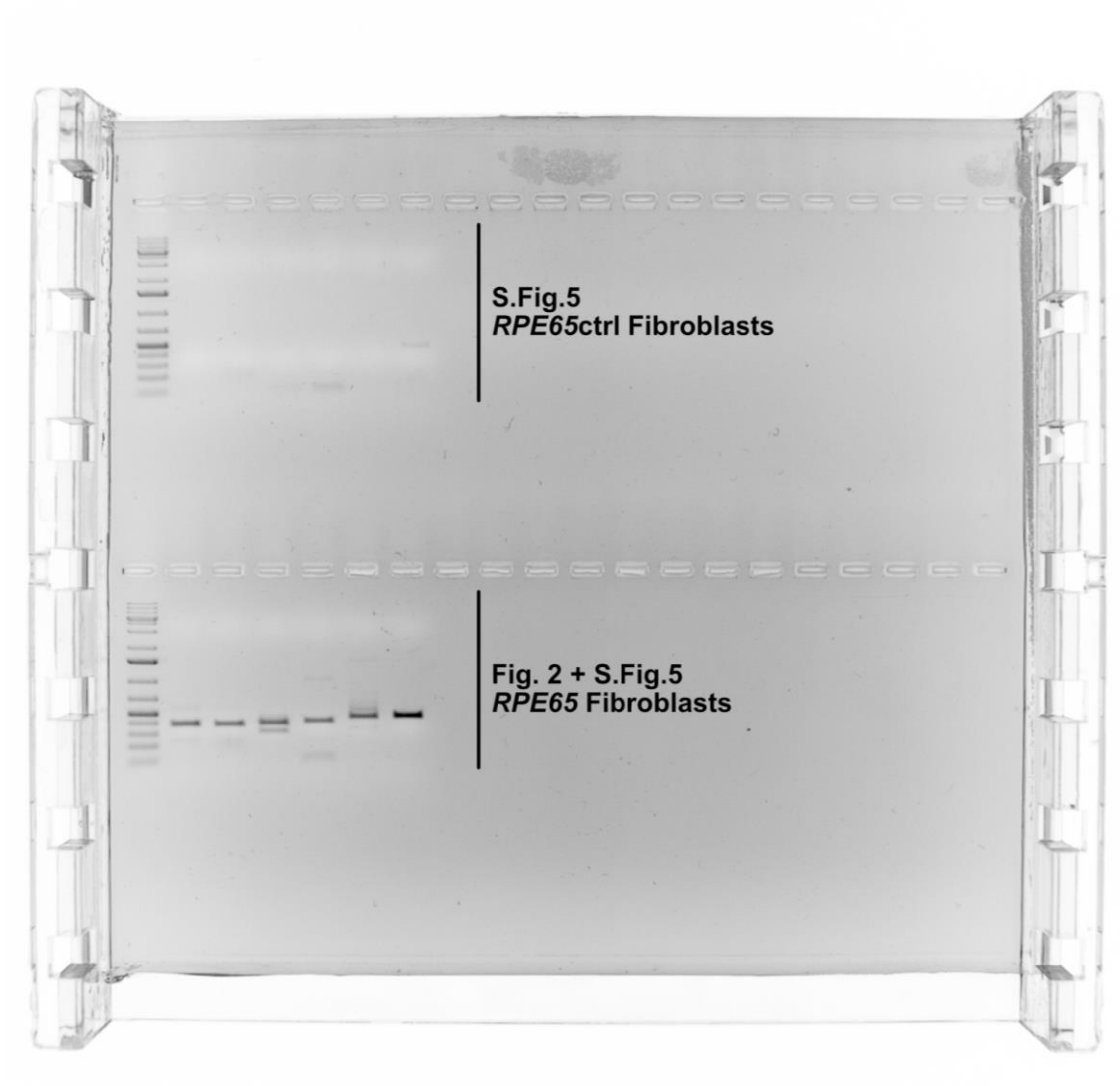

Full unedited gel for Figure 2A and S.Figure 5 (RPE65,PBMC)

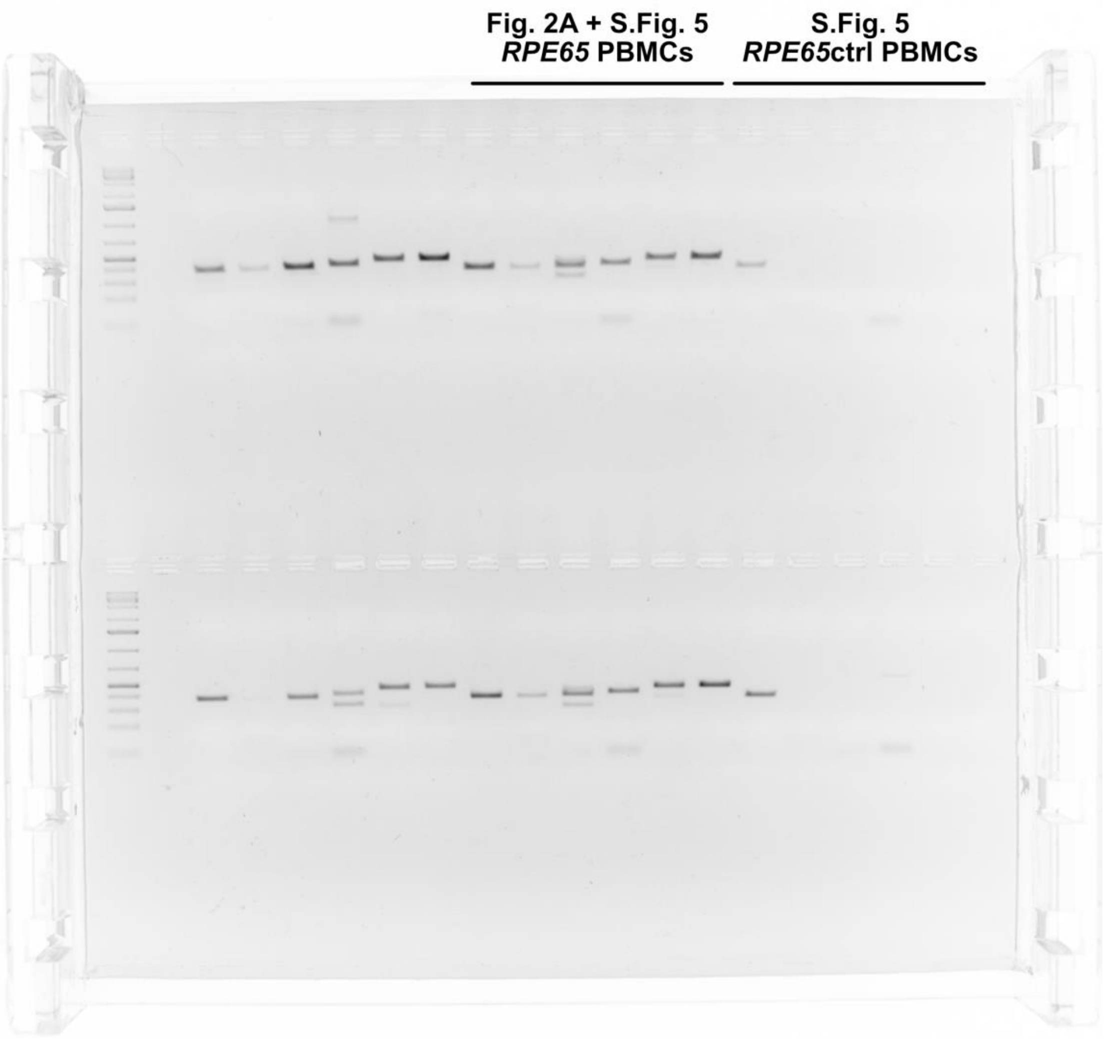

Full unedited gel for Figure 5B (ABCA4_CTRL)

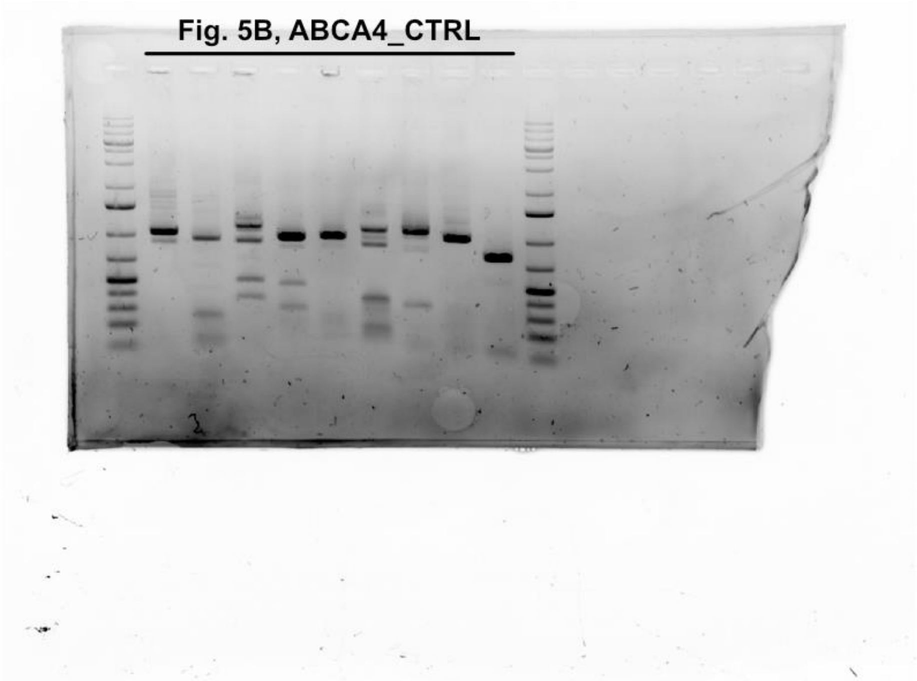

Full unedited gel for Figure 5B and S.Figure 7 (P1 + P1ctrl)

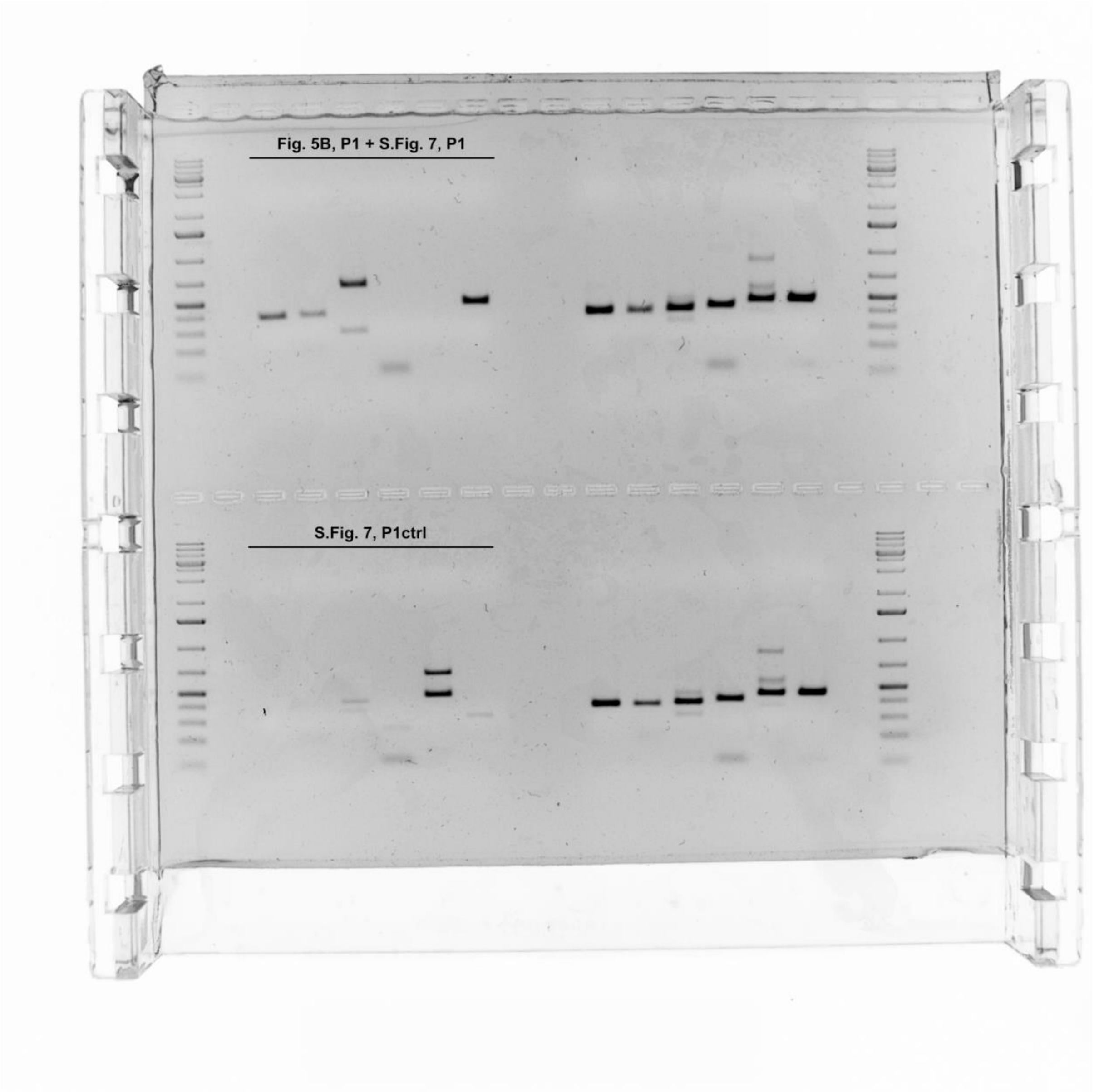

Full unedited gel for Figure 5B and S.Figure 7 (P2, P2ctrl, P3, P3ctrl, RPE65_CTRL)

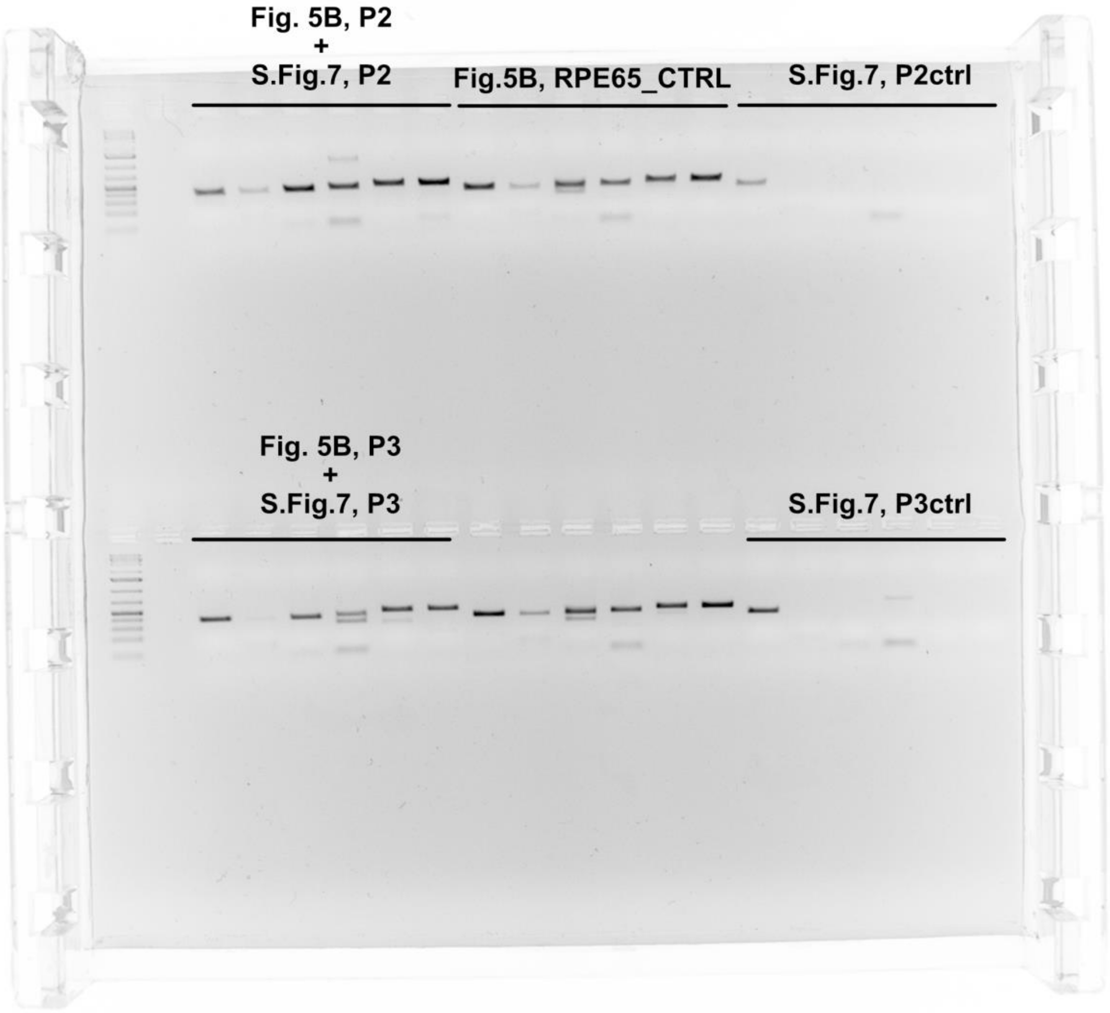

Full unedited gel for Figure 5B and S.Figure 7 (P4)

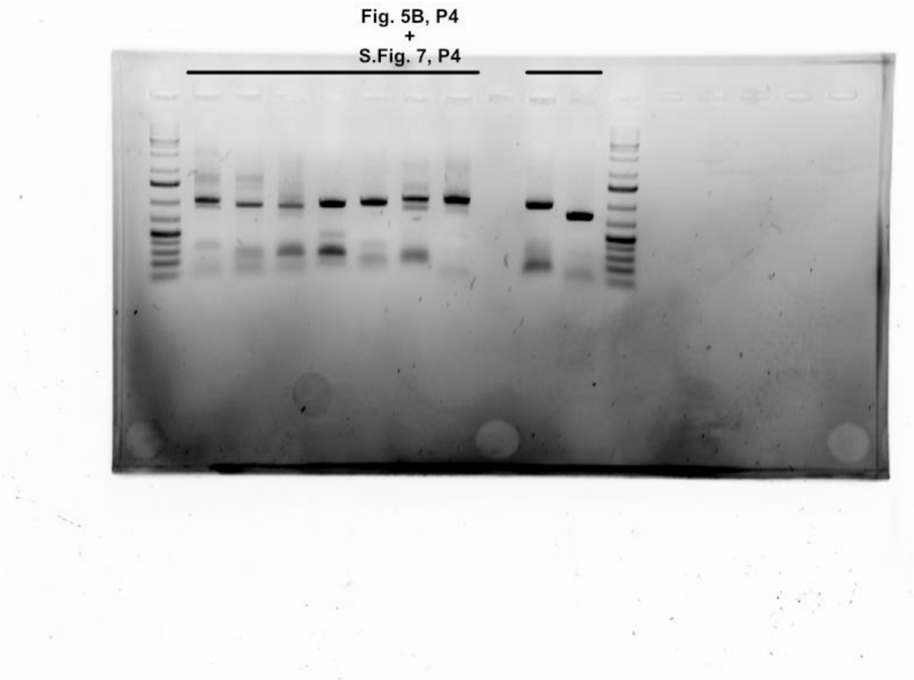

Full unedited gel for Figure 5B and S.Figure 7 (P5)

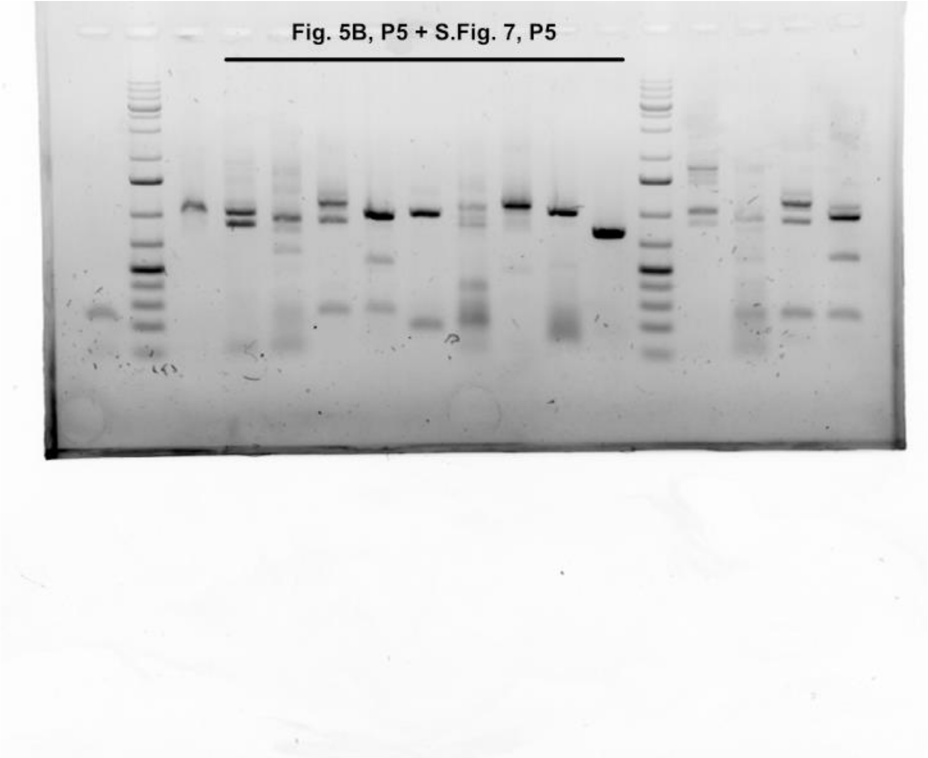

Full unedited gel for Figure 5B and S.Figure 7 (P6 + P6ctrl)

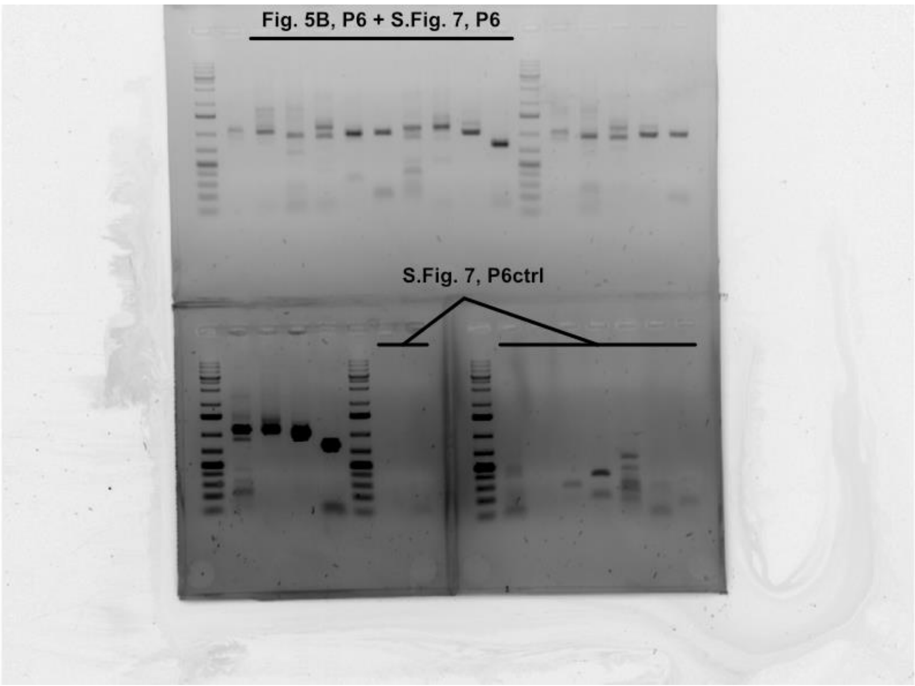

Full unedited gel for S.Figure 4B

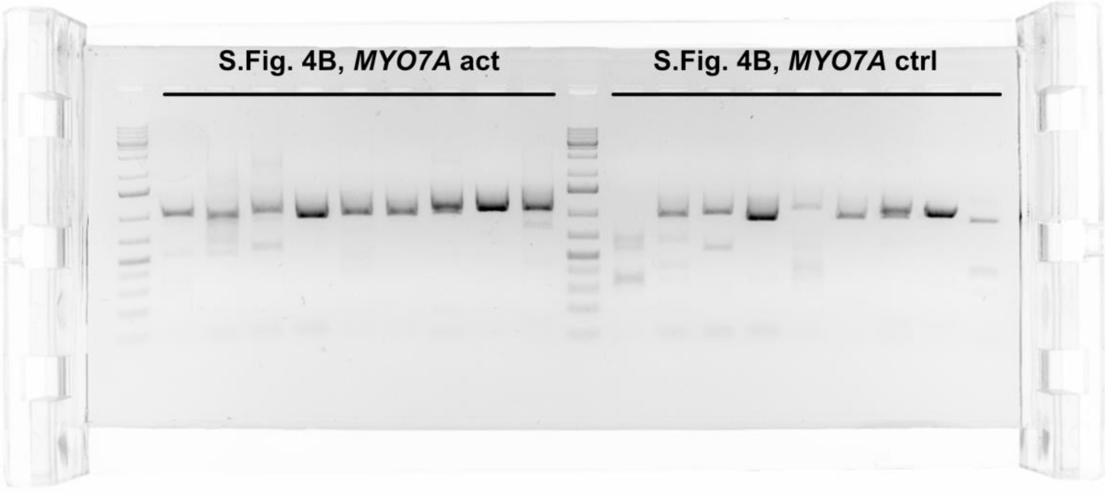

Full unedited gel for S.Figure 4C

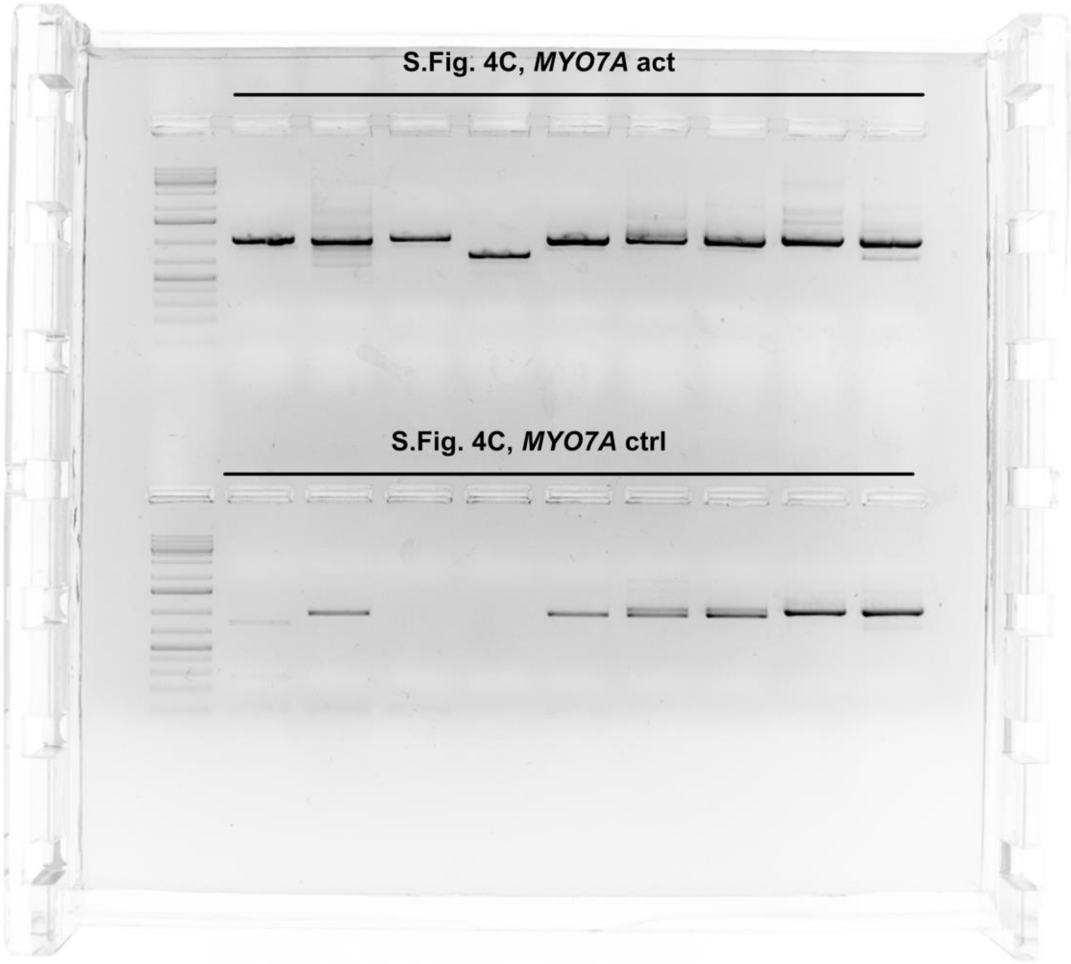

Full unedited gel for S.Figure 4E

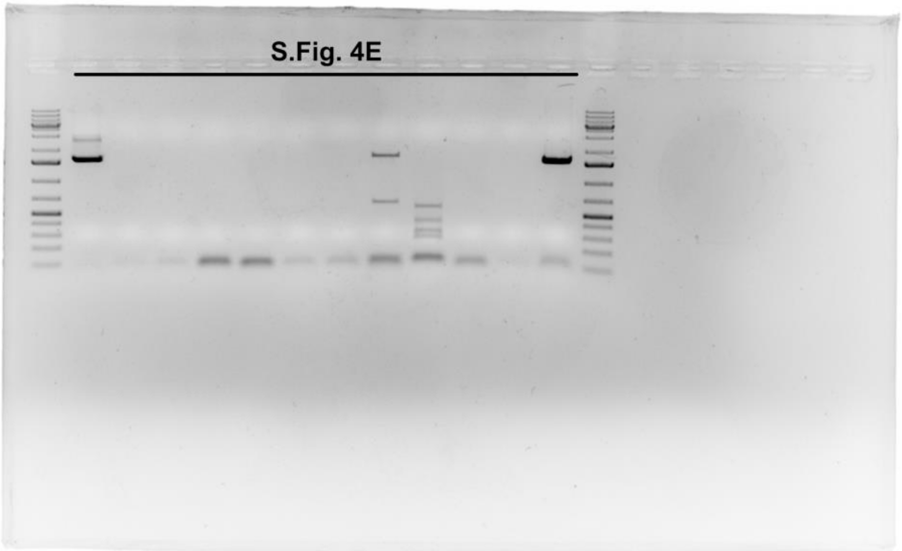

Full unedited gel for S.Figure 5 (ABCA4ctrl,Fibroblast)

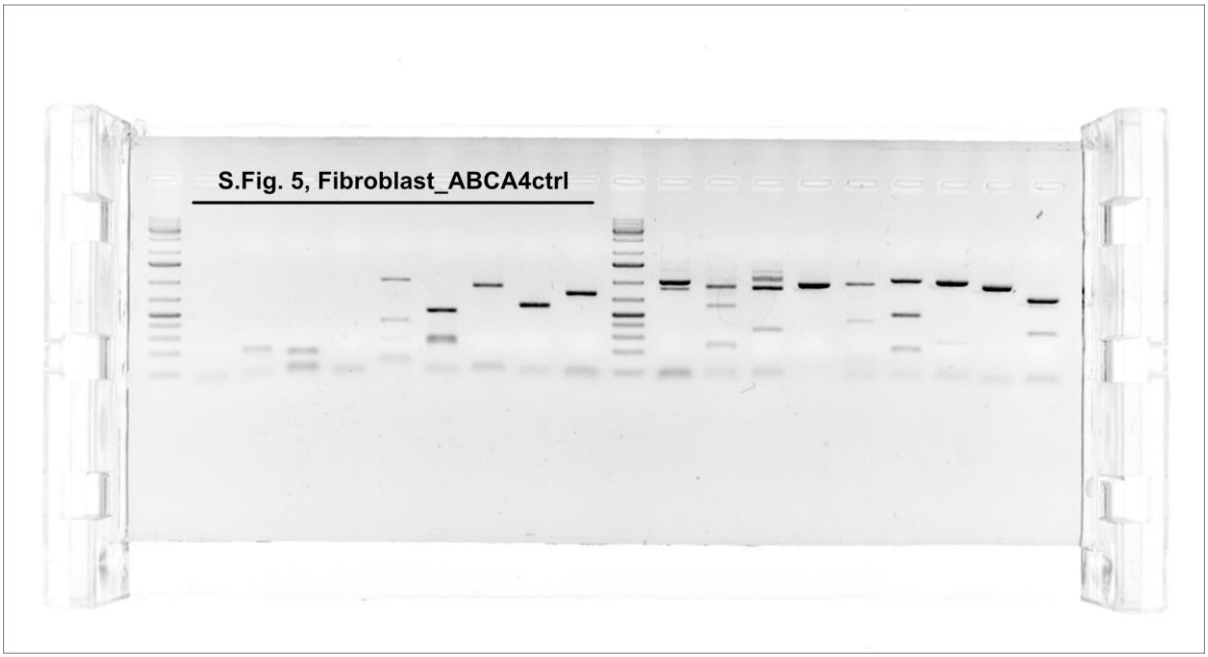

Full unedited gel for S.Figure 5 (ABCA4ctrl,PBMC)

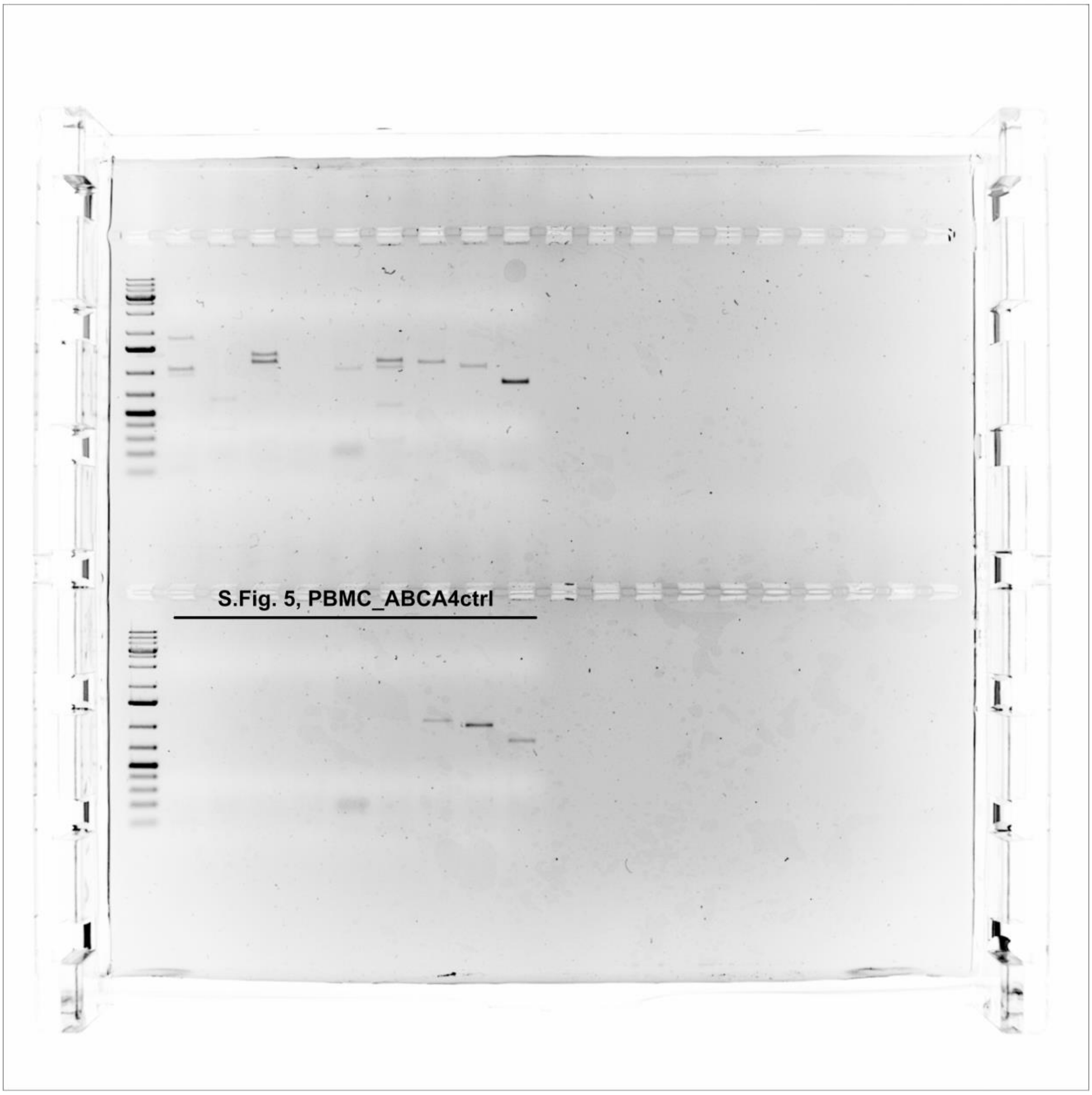

Full unedited gel for S.Figure 6B (USH2A; USH2A_NMDi)

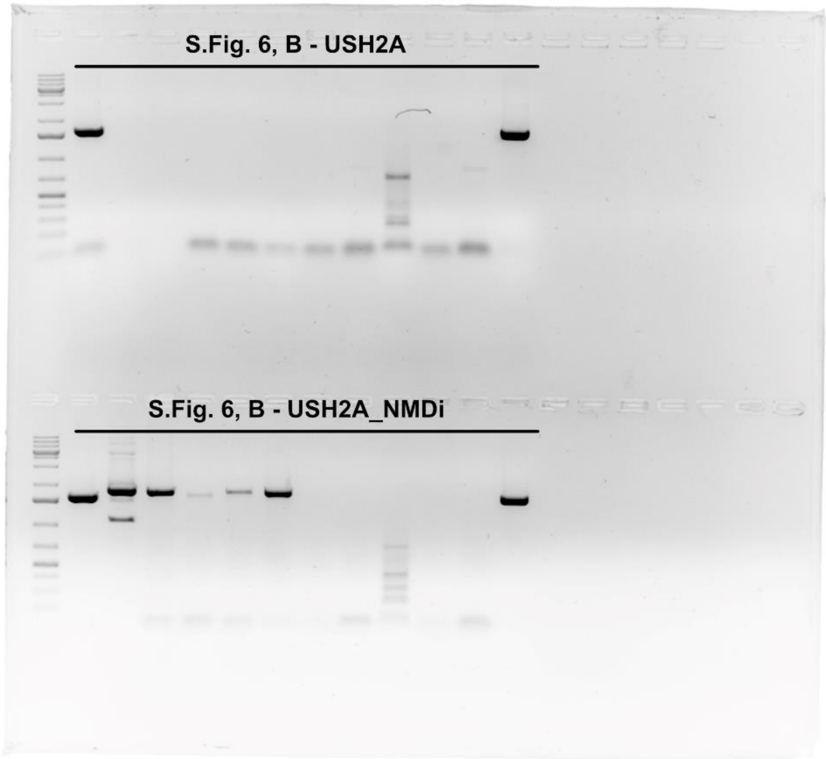

Full unedited gel for S.Figure 6C (USH2A_ST_NMDi)

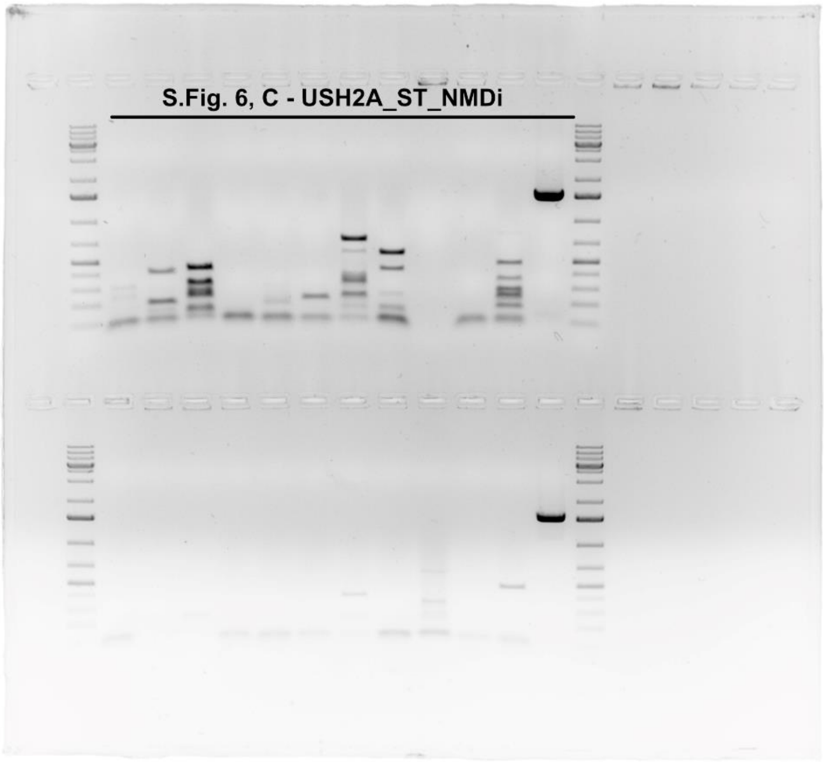

Full unedited gel for S.Figure 6D (HEK293T_USH2A_Suntag)

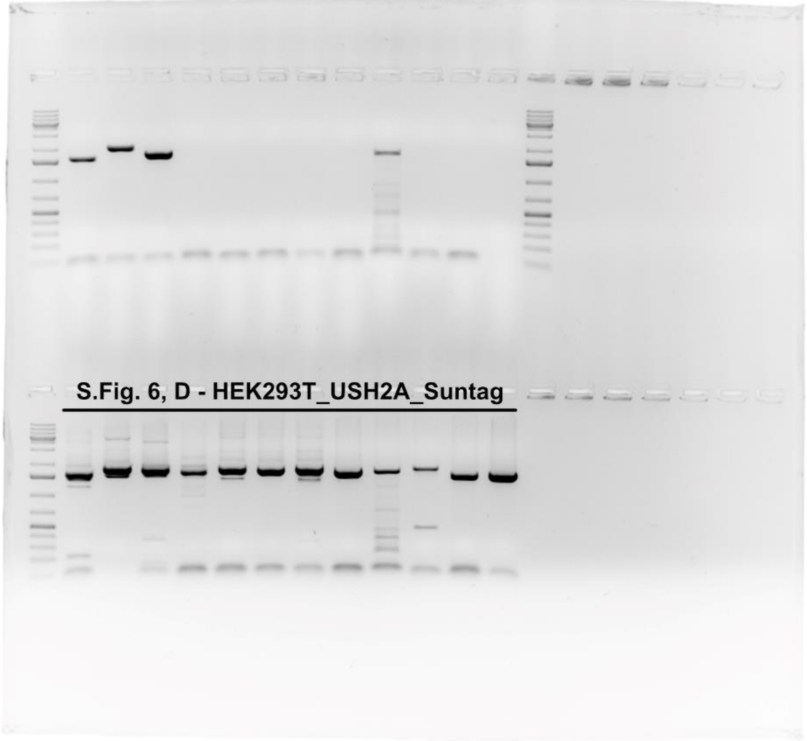

Full unedited gel for S.Figure 6D (HEK293T_USH2A_VPR)

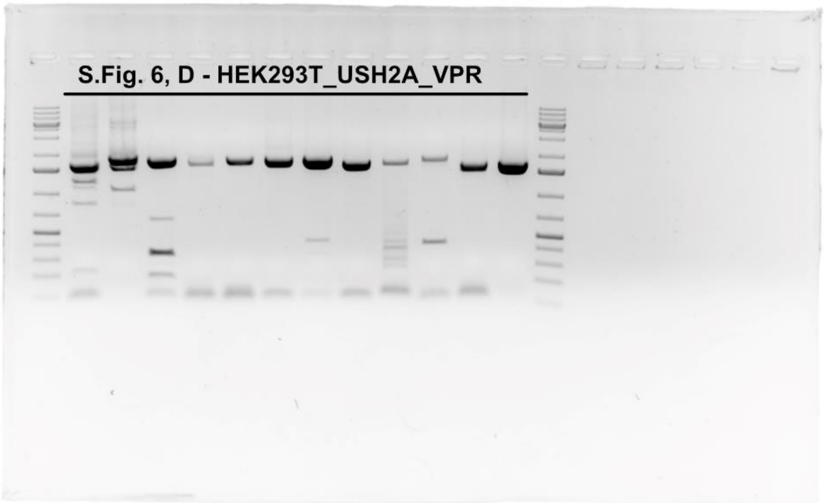

Full unedited gel for S.Figure 9 (P4ctrl)

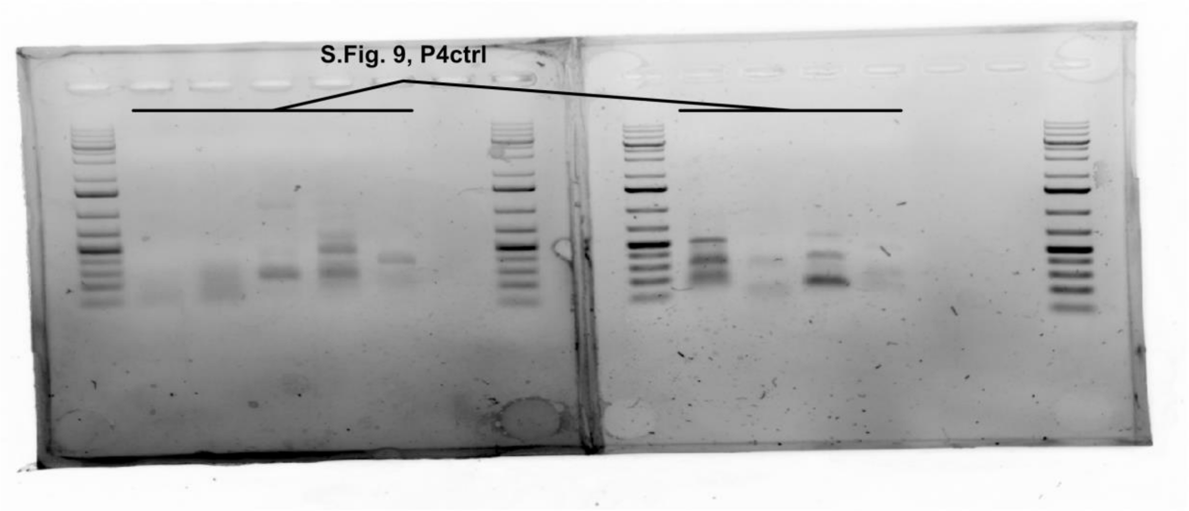

Full unedited gel for S.Figure 7 (P5ctrl)

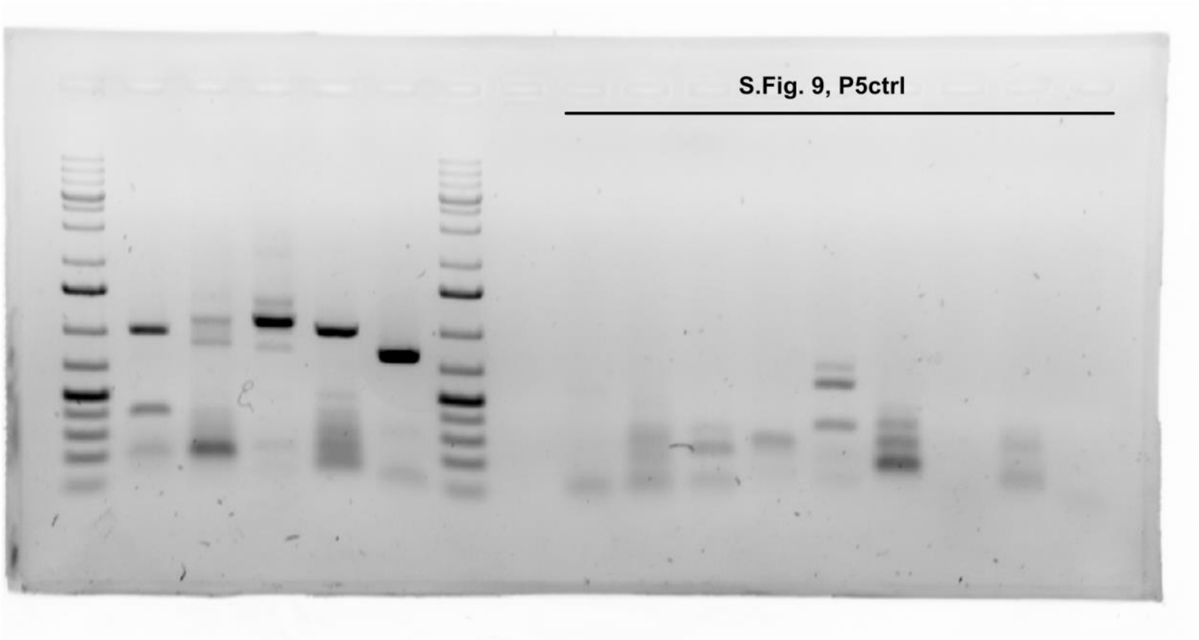

Supplement: Unedited blot and gel images [file jciinsight-10-189615-s234.pdf]
